# Enhanced Antigiardial Effect of Omeprazole Analog Benzimidazole Compounds

**DOI:** 10.3390/molecules25173979

**Published:** 2020-09-01

**Authors:** Beatriz Hernández-Ochoa, Saúl Gómez-Manzo, Adrián Sánchez-Carrillo, Jaime Marcial-Quino, Luz María Rocha-Ramírez, Araceli Santos-Segura, Edson Jiovany Ramírez-Nava, Roberto Arreguin-Espinosa, Miguel Cuevas-Cruz, Alfonso Méndez-Tenorio, Ernesto Calderón-Jaimes

**Affiliations:** 1 Laboratorio de Inmunoquímica, Hospital Infantil de México Federico Gómez, Secretaría de Salud, Ciudad de Mexico 06720, Mexico; ausbir@yahoo.com.mx (A.S.-C.); chelyss68@yahoo.com.mx (A.S.-S.); 2 Laboratorio de Bioquímica Genética, Instituto Nacional de Pediatría, Secretaría de Salud, Ciudad de México 04530, Mexico; saulmanzo@ciencias.unam.mx (S.G.-M.); edsonjiovany@ciencias.unam.mx (E.J.R.-N.); 3 Consejo Nacional de Ciencia y Tecnología (CONACYT), Instituto Nacional de Pediatría, Secretaría de Salud, Ciudad de Mexico 04530, Mexico; jmarcialq@ciencias.unam.mx; 4 Unidad de Investigación en Enfermedades Infecciosas, Hospital Infantil de México Federico Gómez, Secretaría de Salud Dr. Márquez No. 162, Col Doctores, Delegación Cuauhtémoc, Ciudad de México 06720, Mexico; luzmrr7@yahoo.com.mx; 5 Departamento de Química de Biomacromoléculas, Instituto de Química, Universidad Nacional Autónoma de Mexico, Ciudad de Mexico 04510, Mexico; arrespin@unam.mx (R.A.-E.); miguel.ccqi@yahoo.com.mx (M.C.-C.); 6 Laboratorio de Biotecnología y Bioinformática Genómica, Escuela Nacional de Ciencias Biológicas, Instituto Politécnico Nacional, Ciudad de México 11340, Mexico; amendezt@ipn.mx

**Keywords:** omeprazole analogs, giardiasis, inhibition enzyme, giardiacidal compounds

## Abstract

Giardiasis is a diarrheal disease that is highly prevalent in developing countries. Several drugs are available for the treatment of this parasitosis; however, failures in drug therapy are common, and have adverse effects and increased resistance of the parasite to the drug, generating the need to find new alternative treatments. In this study, we synthesized a series of 2-mercaptobenzimidazoles that are derivatives of omeprazole, and the chemical structures were confirmed through mass, ^1^H NMR, and ^13^C NMR techniques. The in vitro efficacy compounds against *Giardia*, as well as its effect on the inhibition of triosephosphate isomerase (TPI) recombinant, were investigated, the inactivation assays were performed with 0.2 mg/mL of the enzyme incubating for 2 h at 37 °C in TE buffer, pH 7.4 with increasing concentrations of the compounds. Among the target compounds, **H-BZM2**, **O_2_N-BZM7**, and **O_2_N-BZM9** had greater antigiardial activity (IC_50_: 36, 14, and 17 µM on trophozoites), and inhibited the TPI enzyme (*K*_2_: 2.3, 3.2, and 2.8 M^−1^ s^−1^) respectively, loading alterations on the secondary structure, global stability, and tertiary structure of the TPI protein. Finally, we demonstrated that it had low toxicity on Caco-2 and HT29 cells. This finding makes it an attractive potential starting point for new antigiardial drugs.

## 1. Introduction

Parasitic infections such as giardiasis are a human health concern worldwide. *Giardia lamblia* is the parasite cause of this human diarrheal disease and is still a major problem in developing countries, mainly affecting the infant population, with high morbidity and mortality indexes due to the effects of severe diarrhea, invasive infections, malabsorption syndrome, and resultant impairment of growth and development [1]. This parasitosis is considered the main cause of epidemic enteritis worldwide, and a re-emerging infectious disease of public health concern [2,3]. Due to the impact of this disease in regions with a poor socioeconomic status and developing countries, in 2004, the World Health Organization (WHO) included giardiasis in the group of neglected tropical diseases [4].

Current pharmacological therapies for giardiasis include metronidazole, tinidazole, ornidazole, and secnidazole; these drugs are derived from the nitroimidazole family, while derivatives of the benzimidazole group include albendazole and mebendazole [5,6,7,8]. All these drugs have different mechanisms of action, involving distinct targets and downstream metabolic pathways and mechanisms that affect cellular processes in trophozoites, such as proliferation and conversion into infective cysts.

Although current drug therapy for the treatment of giardiasis is effective, most available drugs have significant side effects that restrict their use. In addition, the use of metronidazole is seriously undermined by the development of resistance in the target parasite species [9,10,11]. Considering the side effects of the antiprotozoal drugs and the lack of activity of metronidazole against *G. lamblia*, there is an urgent need to develop new drugs to treat giardiasis. In this context, the proton pump inhibitors (PPIs) are extensively used in clinical practice, Omeprazole with other PPIs has been proposed to be potential drugs for the treatment of several diseases including the giardiasis. Has been demonstrated that omeprazole shows cytotoxic effects in *G. lamblia* and concomitantly inactivates the TPI enzyme [12]. The TPI from *G. lamblia*, is an enzyme of the glycolytic pathway of 247 amino acids, with an α/β barrel structure, which catalyzes the isomerization between dihydroxyacetone phosphate (DHAP) and glyceraldehyde-3-phosphate (GAP) [13].

**Figure 1:**
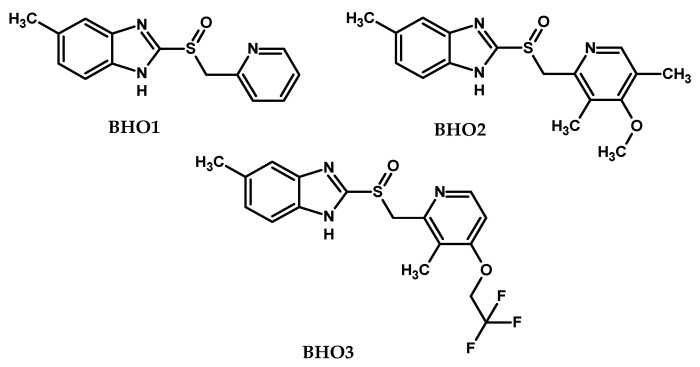
Chemical structure of 2-[(3yridine-2-ylmethyl)sulfinyl]-1*H*-benzimidazole derivatives.

Therefore, we have synthesized compounds with heterocyclic scaffolds bearing different substituents and have found them to be active against the parasite *G. lamblia,* and with the ability to inactivate the TPI. They can be explored as future drug candidates with higher efficacy, resistance effectiveness, and lesser side effects. Among the heterocycles used are benzimidazole, and 2-mercaptobenzimidazoles [14,15], which possess extensive pharmacological effects such as antimicrobial, antihistamine, analgesic, anticonvulsant, and antigiardial activities [16]. In addition, a recent study revealed the synthesis of a series of 2-[(2yridine-2-ylmethyl)sulfinyl]-1*H*-benzimidazole derivatives named **BHO1**, **BHO2**, and **BHO3** (Figure 1) and their antiprotozoal activity against *G. lamblia* was evaluated; two of the evaluated compounds showed antigiardial activity without cytotoxicity in mammalian culture cells [17]. **BHO2** and **BHO3** showed the best antigiardial activity; in addition, it has been reported that one of the pharmacological targets of these compounds is the triosephosphate isomerase from *G. lamblia*. Interestingly, **BHO2** and **BHO3** also have the property of inactivating the TPI enzyme, and it was confirmed by mass spectrometric techniques that the benzimidazole moiety of compounds forms a covalent bond with the cysteine residues present in TPI, which leads to their inactivation.

**Figure 2:**
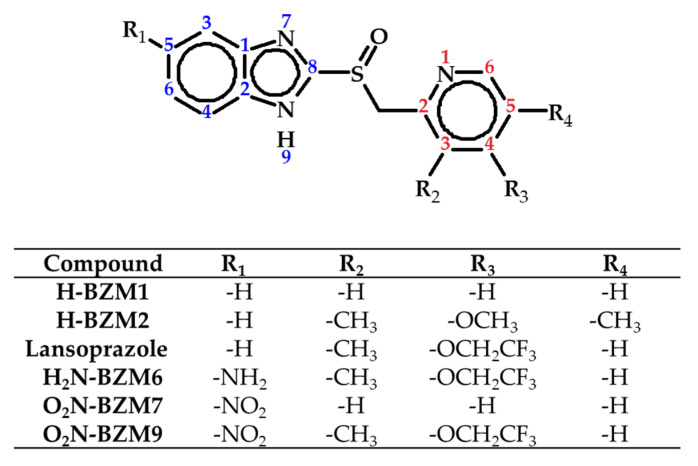
Chemical structure of synthesized 2-mercapto-benzimidazoles, the numbering of the benzimidazole and pyridine core in blue and red color.

Therefore, in this work, we improve the antigiardial activity of new derivatives of 2-mercaptobenzimidazoles with different substituent groups linked to the benzimidazole ring. To reach these aims, five compounds named **H-BZM1**, **H-BZM2**, **H_2_N-BZM6**, **O_2_N-BZM7**, and **O_2_N-BZM9**, (Figure 2), containing the scaffold of proton pump inhibitors but with hydrogen, amino, and nitro substituents groups linked at Position 5 of the benzimidazole ring (R_1_), were synthesized, purified, and characterized, we also perform the synthesis of lansoprazole. In addition, we evaluated the TPI enzyme inactivation ability and the antigiardial capacity of these synthesized compounds. Finally, we demonstrated that **H-BZM2**, **O_2_N-BZM7**, and **O_2_N-BZM9** displayed greater antigiardial activity and low toxicity on Caco-2 and HT29 cells concerning commercial drug omeprazole and the BHO2 compound previously reported. This finding makes an attractive potential starting point for new antigiardial drugs.

## 2. Results and Discussion

### 2.1. Design and Synthesis of Analog Benzimidazoles of Omeprazole

Heterocycle compounds are biologically and structurally important in the fields of chemistry, biochemistry, and medicine. Often, a simple heterocyclic scaffold is utilized for obtaining new biologically active compounds with diverse properties. Recently, it has been reported that 2-mercaptobenzimidazole-derived compounds possess antiparasitic properties [18,19,20]. Interestingly, one of the compounds that has the benzimidazole ring in its structure is omeprazole, which has been studied for its antigiardial properties [12,14,17,21,22]. Furthermore, it has been observed that omeprazole and certain analogs exhibit antigiardial activity, because these compounds act as inhibitors of the glycolytic enzyme triosephosphate isomerase (TPI) of *Giardia lamblia* by covalent binding of benzimidazole moiety with cysteine residues [17], suggesting that this enzyme could be proposed as a potential drug target in the antigiardial activity of these compounds [12]. Based on the above, and in order of increase, the antigiardial activity of 2-mercaptobenzimidazole-derived compounds, five analogs (**H-BZM1**, **H-BZM2**, **H_2_N-BZM6**, **O_2_N-BZM7**, and **O_2_N-BZM9**) with different substituent groups linked to the benzimidazole ring was designed and synthesized. It is important to note that the synthesis of the **O_2_N-BZM7** compound, previously was described in Patent Number 4,791,114 [23]; however, in the present work, a different synthesis method was used to obtain the **O_2_N-BZM7** compound. Besides, the synthesis of the lansoprazole drug was performed to also be evaluated [24,25,26]. In Position 5 of the benzimidazole core (R_1_), we placed a hydrogen atom in compounds **H-BZM1** and **H-BZM3** (Figure 2), as well as lansoprazole, while an amine group was placed in the **H_2_N-BZM6** compound. Finally, a nitro group was placed in both the **O_2_N-BZM7** and **O_2_N-BZM9** compounds in the same position (Position 5 of the benzimidazole core (R_1_)). The changes performed in the pyridine ring were as follows: no substituents (R_2_–R_4_) for **H-BZM1** and **O_2_N-BZM7**; **H-BZM2** has a pyridine ring substituted with two methyl groups (R2 and R4), and a methoxy group (R_3_); and **H_2_N-BZM6**, and **O_2_N-BZM9** possess a pyridine with substituents of lansoprazole a methyl group (R_2_) and trifluoroethoxy group (R_3_; Figure 2).

**Scheme 1:**
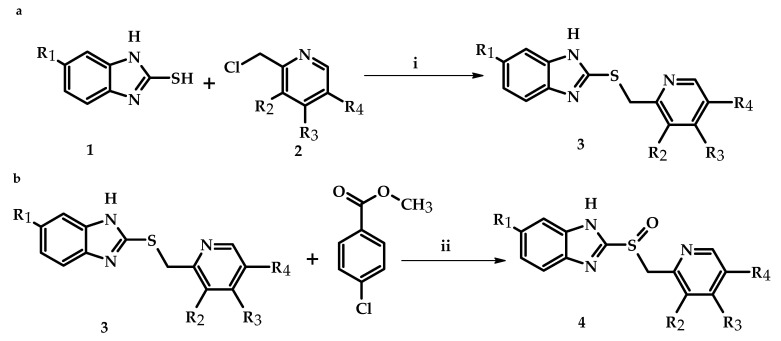
Syntheses of compounds. The synthesis was performed through a two-step method. (**a**) Obtaining intermediaries, prepared by nucleophilic substitutions between the 2-mercapto-5-methylbenzimidazole with one of the substituted 2-chloromethy pyridine, in an alkaline medium. (**b**) Sulfoxide synthesis. Oxidation of thioether group with a peroxide acid.

Syntheses of compounds were carried out as previously reported by Hernández-Ochoa et al. [17]. Briefly, the synthesis was in two steps, as shown in Scheme 1. First, the precursors were prepared by nucleophilic substitutions between the 2-mercapto-5-methylbenzimidazole with one of the substituted 2-chloromethy pyridine and in the presence of potassium hydroxide, at 50 °C for 8 h, which gave the desired the 5-methyl-2-[(4yridine-2-ylmethyl)sulfanyl]-1*H*-benzimidazole compound. Then, the precursors were purified by silica gel column chromatography and their chemical structures confirmed by NMR spectroscopy. Second, oxidation of the thioether group in precursors was done to obtain the sulfoxide products by using peroxide acid (Scheme 1), in this step, it was important to keep the reaction at 0 °C and to a slow addition of the peroxide acid, which allowed to obtain the desired final sulfoxides and avoid the formation of the sulfone. Afterward, the final compounds were purified by silica gel column chromatography and their chemical structures confirmed by NMR spectroscopy (see Figures S1, S2, S4, S5, S7, S8, S10, S11, S13, and S14 in Supplementary Materials). The compounds were stored in perfectly closed amber flasks at 4 °C, protected from light until later use, to avoid decomposition. The stock solutions of each of the compounds were prepared immediately before the experiments.

The compounds were obtained with yields between 70% and 85% as brown solids with similar melting points. The ^1^H NMR and ^13^C NMR spectra of all final synthesized compounds and precursors are in total agreement with the suggested structures (Figure 2 and Scheme 1). Additional support for the proposed structures comes from mass spectral data obtained from both low- and high-resolution mass spectra of the synthesized compounds showing the correct molecular ions, (M^+^), as suggested by their molecular formulas or fragments that stem from the molecular ion. Analysis of the molecular ions and the fragmentation pattern are used in the identification and characterization of these compounds (Figures S3, S6, S9, S12, and S15 in Supplementary Materials).

### 2.2. Dose-Dependent Effect of New Compounds on Giardia lamblia

**Figure 3:**
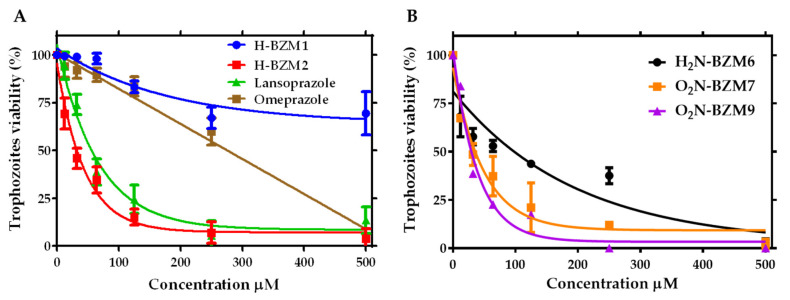
Antigiardial activity of the five novels synthesized compounds and lansoprazole on *G. lamblia* trophozoites’ viability. The trophozoites were incubated in the presence of (**A**) **H-BZM1**, **H-BZM2** compounds, and **lansoprazole**; (**B**) **H_2_N-BZM6**, **O_2_N-BZM7**, and **O_2_N-BZM9** compounds by 48 h at 37 °C with increasing concentrations of each of the compounds, and later the viability of the trophozoites were determined. The values represent mean ± standard deviation from three independent experiments, standard errors were lower than 5%.

In order to determine the antigiardial effects of each of the synthesized compounds and the lansoprazole, we used dose-dependent curves to determine the percentage of viable trophozoites from *G. lamblia* after incubation at different concentrations of each of the compounds using a trypan blue dye exclusion assay. The mechanism of action of omeprazole and other PPIs has been studied, and it is documented that in *G. lamblia* the efficacy and species-specific action of omeprazole to inhibit TPI is possible under an acidic or neutral pH [12,17]. Based on the above, in the present study, the cytotoxicity and inactivation assays were carried out at pH 7.4 and 37 °C. As seen in Figure 3, all compounds evaluated here inhibited the viability of *G. lamblia* trophozoites to different extents. The inhibitory effects of the synthesized compounds on the viability of the parasite are shown in Figure 3, where a dose-dependent inhibition was observed. Then, the IC_50_ values (the concentration of compound that is required to kill 50% of trophozoites in vitro) were calculated. For the compounds with a hydrogen atom at Position 5 in the benzimidazole ring (R_1_), the IC_50_ values calculated were 676 µM for **H-BZM1**, 36 µM for **H-BZM2**, and 45 µM for lansoprazole, respectively (Figure 3A and Table 1); **H_2_N-BZM6** with an amino group at the same position inhibited parasite viability and had an IC_50_ value of 135 µM (Figure 3B). The maximal inhibitory effects were observed with compounds that have a nitro group in the benzimidazole ring; IC_50_ values were 14 µM and 17 µM for **O_2_N-BZM7** and **O_2_N-BZM9**, respectively. It is important to note that **H-BZM2**, lansoprazole, **O_2_N-BZM7**, and **O_2_N-BZM9** showed an increased cytotoxic effect on *G. lamblia* trophozoites from 2.6- to 8.5-fold compared with that previously reported for **BHO2** derived from omeprazole, where an IC_50_ value of 120 µM was determined [17]. These results suggest that the five compounds are more active against *Giardia* trophozoites. Based on the above, we observe that, despite the structural similarity concerning the ring pyridine between compounds lansoprazole, **H_2_N-BZM6**, and **O_2_N-BZM9**, they showed differences in their biological activities, which indicates the importance of the substituents on the benzimidazole ring. According to this, compounds lansoprazole and **O_2_N-BZM9** exhibited approximately three- to eight-fold higher activity than **H_2_N-BZM6** against *G. lamblia*. This increase in the antigiardial effect obtained with the **O_2_N-BZM9** compound is probably due to the presence of the nitro group, which could be involved in the redox metabolism, affecting the survival of the trophozoites in a similar pathway to the mechanism observed with metronidazole (MTZ) [13]. Additionally, the experimental results showed that in the compounds with no substituted pyridine (**H-BZM1** and **O_2_N-BZM7**), the presence of a nitro group at Position 5 of the benzimidazole nucleus (R_1_) is essential for increased antigiardial activity, explaining why compound **H-BZM1** is 48-fold less active than **O_2_N-BZM7** in terms of the antigiardial effect.

### 2.3. Cytotoxic Effect of Compounds on Human Intestinal Caco-2 and HT29 Cells

**Figure 4:**
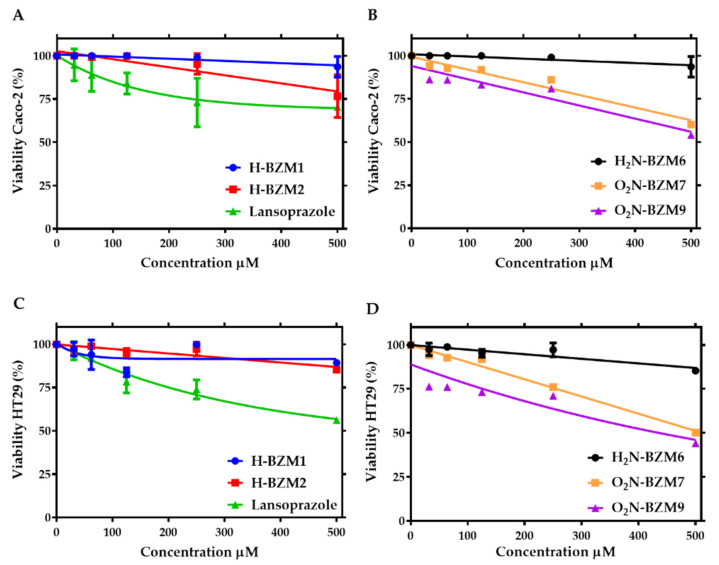
Cytotoxic effect of compounds on Human Intestinal Caco-2 and HT29 cells Dose‒response curves for cell viability of (**A**) Caco-2 cells and (**B**) HT29 cells treated with **H-BZM1**, **H-BZM2**, and **H-BZM3**. Dose‒response curves for cell viability (**C**) Caco-2 cells and (**D**) HT29 of HT29 cells treated with **H_2_N-BZM6**, **O_2_N-BZM7**, and **O_2_N-BZM9**. The viability was determined by using an XTT assay. The values represent the mean ± standard deviation from three independent experiments, standard errors were lower than 5%.

One of the main problems in the development of new drugs is the high toxicity to mammalian cells. Therefore, we performed an assay to determine the cytotoxic effect of the six novel synthesized compounds with antigiardial activity on two mammalian cell lines. For this, we used the cultures of Caco-2 and HT29 cells as a model of the intestinal epithelium. By XTT (2,3-Bis(2-methoxy-4-nitro-5-sulfophenyl)−2*H*-tetrazolium-5-carboxamide) assay, we identified the concentration-dependent effects on Caco-2 and HT29 cells’ viability of all compounds, and the CC_50_ values (the concentration of the compounds that killed 50% of the cells) were determined as shown in Table 1. The results demonstrate that **H-BZM2** and lansoprazole had low toxicity towards Caco-2 and HT29 eukaryotic cells, even at the highest concentrations tested (500 µM); a cell viability decrease of approximately 20% was seen in Figure 4. It is interesting to note that these same two compounds (**H-BZM2** and lansoprazole) have a good antigiardial effect against parasites, with IC_50_ values of 36 µM and 45 µM for **H-BZM2** and lansoprazole, respectively. These results are important because they indicate that these compounds could be proposed as new antigiardial drugs for further studies.

Concerning the **O_2_N-BZM7** and **O_2_N-BZM9** compounds, we observed a negative effect on Caco-2 and HT-29 mammalian cells, lowering the viability to almost 50% when the cells were treated with 500 µM of these compounds. However, at 150 µM, where the viability of the *G. lamblia* trophozoites was abolished at 100% (Figure 3B), we observed that at the same concentration Caco-2 and HT29 mammalian cells’ viability was decreased by around 20% (Figure 4C,D).

The selectivity index (SI) demonstrates the differential activity of a pure compound: the greater the SI value, the more selective it is; thus, it is desirable to have a high SI, giving maximum antigiardial activity with minimal cell toxicity. Based on the SI, the data shown in Table 1 indicate that the compounds exhibit a high degree of cytotoxic selectivity on *G. lamblia*. We propose that **H-BZM2**, lansoprazole, **O_2_N-BZM7**, and **O_2_N-BZM9** be considered for follow-up as antigiardial candidates with minimal cell toxicity. These results are in concordance with those previously observed in VERO cells, where the compound **BHO2** exhibited CC_50_ values for mammal cells in the millimolar ranges, which are 38- to 61-fold higher concerning the IC_50_ obtained for trophozoites [17].

### 2.4. In Vitro Screening of Triosephosphate Isomerase Inactivation

As we observed antigiardial activity of all the novel synthesized compounds on *G. lamblia* trophozoites, we decided to evaluate in vitro the effect of the compounds in the inactivation TPI enzyme from *G. lamblia*.

**Figure 5:**
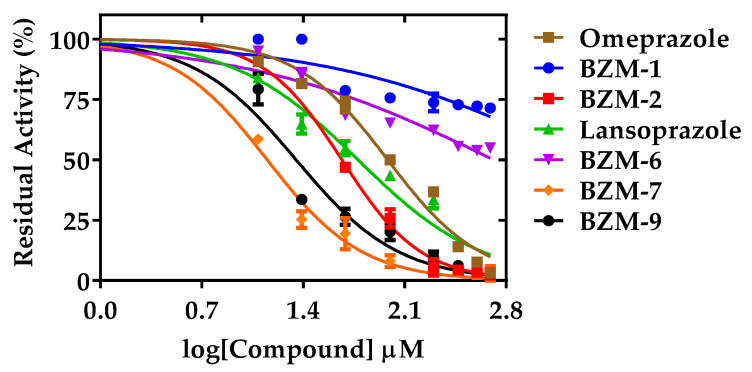
Inactivation assays of triosephosphate isomerase (TPI) enzyme from *G. lamblia* with the compounds. TPI protein was adjusted at a final concentration of 200 μg/mL and incubated with increasing concentrations of each of the compounds. After 2 h of incubation at 37 °C, the reactions were stopped by dilution, and the remaining activity was determined as described in Section 3.5 of Materials and Methods. The figure shows representative experiments performed in triplicate. The values represent the mean ± standard deviation from three independent experiments, standard errors were lower than 5%.

Previously, it was reported that both omeprazole and certain analogs such as **BHO2** have as a target in nonconservative Cys residue at Position 222 of glycolytic TPI enzyme from *G. lamblia*, although this residue is not near the catalytic site [12,17,21]. Hence, it is expected that these novel synthesized compounds will affect the TPI activity of *G. lamblia* by derivatization cysteines but in a more efficient form. We performed an inactivation assay to evaluate the effect of compounds on the TPI activity using different concentrations of these novel synthesized compounds. Figure 5 shows the residual activity of the TPI enzyme after incubation for 2 h at 37 °C. As the concentration of each of the compounds was increased, the residual TPI activity decreased for all the compounds. The exposure of the TPI enzyme to **H-BZM2**, lansoprazole, **O_2_N-BZM7**, and **O_2_N-BZM9** induced the abolition of enzyme activity in a very similar manner. The TPI enzyme lost 100% of its activity when incubated with these compounds at 500 µM. In contrast, **H-BZM1** and **H_2_N-BZM6** scarcely inactivated the TPI enzyme, with 25% and 40% reductions, respectively, at the highest concentration (500 µM). From the plots of residual activity data, we calculated the IC_50_ values; the efficacy of **H-BZM2**, lansoprazole, **O_2_N-BZM7**, and **O_2_N-BZM9** was particularly remarkable because the concentrations required to reduce the activity of the TPI enzyme by 50% were 37 µM, 66 µM, 12 µM, and 20 μM, respectively (Figure 5). It is interesting to note that, of the five synthesized compounds, three of them, **H-BZM2**, **O_2_N-BZM7**, and **O_2_N-BZM9**, and lansoprazole led to increased inactivation of the TPI enzyme from *G. lamblia* concerning **BHO2** and omeprazole, previously reported [17]. As described before, **BHO2** affects the activity of the TPI enzyme, with IC_50_ of 64 µM [17], where this compound has a methyl group Position 5 in the benzimidazole ring (R_1_). In the results obtained here, we observed that with the substitution of a hydrogen atom in Position 5 of the benzimidazole ring (R_1_), we were able to enhance the inactivation of the TPI enzyme with compound **H-BZM2** and the same result was observed with lansoprazole which also has a hydrogen. However, when a nitro group is placed in that same position, the compound shows a high ability to inactivate the TPI enzyme since **O_2_N-BZM7** and **O_2_N-BZM9** show IC_50_ values of 12 µM and 20 µM, respectively, with enhancements of 5.3-fold (**O_2_N-BZM7**) and 3.2-fold (**O_2_N-BZM9**) observed with respect to **BHO2**.

Finally, It is interesting to note that compounds **H-BZM2**, lansoprazole, **O_2_N-BZM7**, and **O_2_N-BZM9** had a good inhibitory effect on the viability of *G. lamblia*, and that these compounds did not affect the viability of human intestinal cells, making them good candidate antigiardial drugs. Probably, the inhibitory effect on the viability could be due to TPI inactivation in trophozoites, as was demonstrated recently by López-Velazquez et al. [27], who demonstrated that omeprazole targets and inhibits recombinant GlTIM (TPI from *G. lamblia*) in bacterial cells.

### 2.5. Second-order Inactivation Constant (k_2_) of TPI Exposed to Selected Hit Compounds

**Figure 6:**
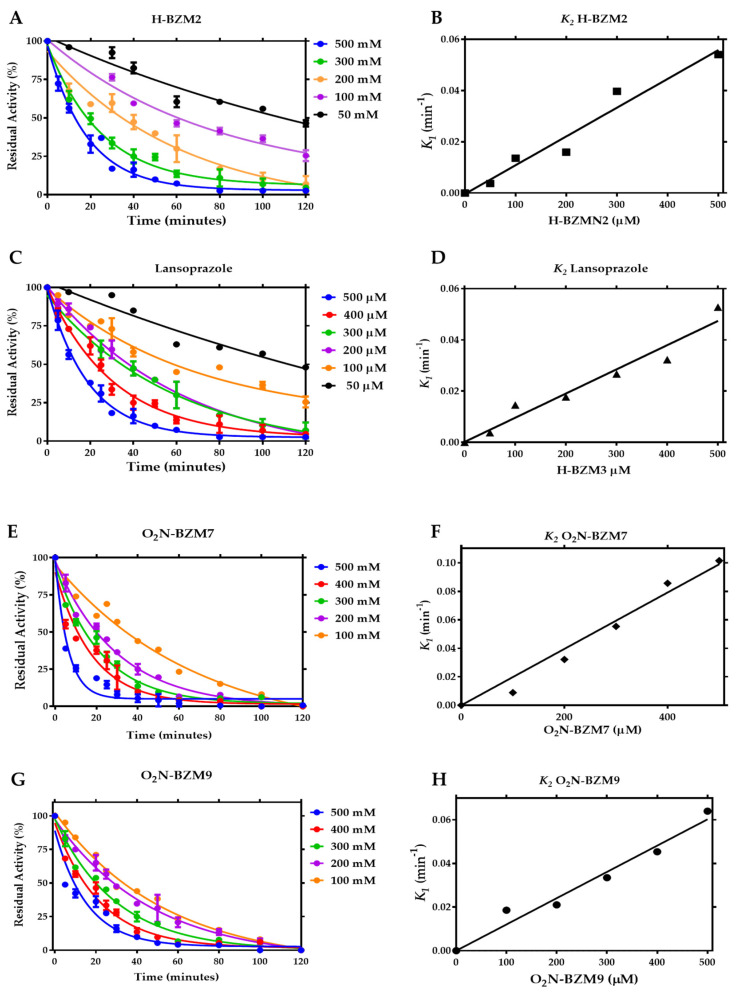
Inactivation assays of the TPI enzyme from *G. lamblia* with **H-BZM2**, lansoprazole, **O_2_N-BZM7**, and **O_2_N-BZM9**. (**A**,**C**,**E**,**G**) The pseudo-first-order rate constant values (*k*_1_) for each compound were calculated by fitting the data to the exponential decay equation *A* = *A*_0_*e-^k1t^*, where *A*_0_ is the initial activity value. All experiments were performed in triplicate; the bars represent the standard error. (**B**,**D**,**F**,**H**) The second-order rate constant values of inactivation (*k*_2_) were obtained by plotting the *k*_1_ values against the concentrations of compounds and adjusted to a linear regression model. All the experiments were performed in triplicate; standard errors were lower than 5%.

To compare the reactivity of five compounds and the lansoprazole on the TPI enzyme with respect to **BHO2** and omeprazole, we calculated the rate constants of inactivation. The second-order inactivation constant *k*_2_ (M^−1^ s^−1^) represents the rate of formation of the enzyme‒inhibitor complex. First, we determine the pseudo-first-order inactivation constants (*k*_1_) after the enzyme was incubated with different fixed concentrations of **H-BZM2**, lansoprazole, **O_2_N-BZM7**, and **O_2_N-BZM9**, and the initial velocities were determined at different incubation times. Figure 6 shows the behavior of the four best-synthesized compounds, where a single-exponential decay of time-course inactivation was observed (from 0 to 120 min). This behavior indicates that the inhibitor‒enzyme complex forms rapidly. The *k*_1_ values for each compound were calculated and plotted against their concentrations (Figure 6), and a linear behavior was obtained and fitted with the linear equation. The calculated *k*_2_ values for **H-BZM2** and lansoprazole compounds were 2.3 M^−1^ s^−1^ and 1.78 M^−1^ s^−1^, respectively (Figure 6B,D); these results confirm that a hydrogen atom at Position 5 on the benzimidazole ring (R_1_) is important in order to improve the inactivation of the TPI enzyme from *G. lamblia*. Furthermore, it is interesting to note that these results are in accordance with the *k*_2_ value reported by García-Torres et al. [12] for rabeprazole (*k*_2_ = 2.66 M^−1^ s^−1^), which also contains a hydrogen atom on the benzimidazole ring (R_1_). However, we managed to obtain two compounds that inactivate TPI more powerfully; this was observed with the **O_2_N-BZM7** and **O_2_N-BZM9** compounds, which showed *k*_2_ values of 3.2 M^−1^s^−1^ and 2.8 M^−1^s^−1^, respectively (Figure 6F,H). These results suggest that, by placing an electroattractor substituent, such as a nitro group, on the benzimidazole ring (R_1_), an increase in the inactivation of the TPI enzyme from *G. lamblia* can be obtained.

The values of the second-order rate constant for synthesized compounds (**H-BZM2**, **O_2_N-BZM7**, and **O_2_N-BZM9**) and lansoprazole are higher than omeprazole (0.78 M^−1^ s^−1^) and **BHO2** (1.6 M^−1^ s^−1^), whose second-order rate constant values were previously reported [17]. Our results show a high ability of the novel synthesized compounds to form enzyme‒inhibitor complexes with the TPI enzyme from *G. lamblia*. It is interesting to note that the four synthesized compounds showed an increase from 2.3- to 5.6-fold with respect to omeprazole, and an increase from 1.1- to 1.6-fold was obtained with respect to **BHO2**. Thus, changes in the benzimidazole ring substantially improved the inactivating reactivity of compounds against the TPI enzyme from *G. lamblia.*

### 2.6. Structural Studies of TPI Enzyme with the Inhibitory Compound

#### 2.6.1. Circular Dichroism (CD) Assay

**Figure 7:**
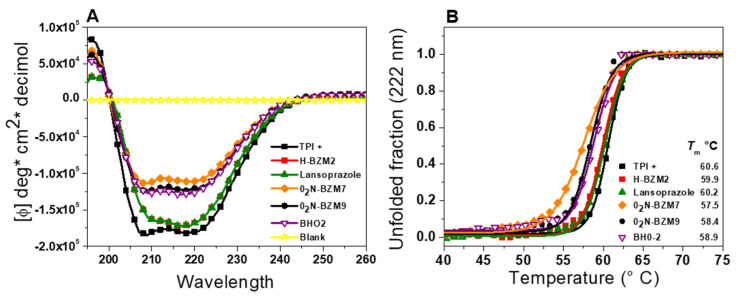
Spectroscopic characterization of TPI from *G. lamblia*. (**A**) Far-UV circular dichroism (CD) spectra and (**B**) thermal stability of the TPI protein. Changes in molar ellipticity were monitored by circular dichroism when the temperature was increased progressively from 35 to 70 °C at 1 °C/2.5 min. In both assays, the protein was adjusted to 0.2 mg/mL in 50 mM phosphate buffer (buffer P) and incubated with any of the four compounds at the IC_50_ concentration of each inhibitor for 2 h at 37 °C prior to measurement. The experiment is representative of triplicate experiments.

To analyze the effect of the synthesized compounds and lansoprazole on the secondary structure of the TPI protein, circular dichroism (CD) assays were performed in the absence (free of compound) or presence of the IC_50_ concentration after incubating the enzyme with each of the compounds. As seen in Figure 7A, the spectra of the TPI enzyme from *G. lamblia* in the absence of the compounds showed minimum absorption peaks at 208 nm and 222 nm, corresponding to β-sheets and α-helices, as previously noted [17]. However, when the TPI enzyme was incubated with the three synthesized compounds and lansoprazole, differences were observed in the minimum absorption signals with respect to the enzyme free of treatment. The compounds that most altered the secondary structure were **O_2_N-BZM7** and **O_2_N-BZM9** because these compounds showed spectra closer to the blank. These results are in agreement with those previously obtained in inactivation assays, which revealed that these two compounds lowered the activity of the enzyme more efficiently. Furthermore, it is interesting to note that these compounds showed a higher alteration of the secondary structure of the TPI enzyme from *G. lamblia* with respect to **BHO2**, as previously reported [17]. **H-BZM2** and lansoprazole showed less of an alteration in the secondary structure of the TPI enzyme, and the signals related to the β-sheets were the most altered. These results indicate that the four compounds affect the secondary structure of the TPI enzyme from *G. lamblia*, which explains the loss of catalytic activity and is in concordance with the results of biochemical assays in which it was detected that the compounds **H-BZM2**, **O_2_N-BZM7**, **O_2_N-BZM9**, and lansoprazole were fastest at forming enzyme‒inhibitor complexes and had a good inhibitory effect on the viability of *G. lamblia*.

#### 2.6.2. Thermal Stability Assays

Because the compounds and lansoprazole reduce the catalytic activity and alter the secondary structure of the TPI enzyme from *G. lamblia*, we evaluated the effect on the thermal stability ™ of the TPI enzyme after incubation with the three compounds and the lansoprazole, following changes in the CD signal at 222 nm measuring the unfolded fraction of the α-helices through a temperature gradient (35–75 °C). As seen in Figure 7B, in the denaturation profiles of TPI free of treatment and incubated with the compounds, we observed that the protein remains in its native structure around 50 °C (baseline); above this temperature, the protein was denatured and completely unfolded at approximately 65 °C. From the plot, the *T*_m_ value (temperature at which half the α-helices remain in the native state and the other half are in an unfolded state) calculated for the TPI free of treatment was 60.6 °C, while for the **H-BZM2** and lansoprazole the *T*_m_ values determined were 59.9 °C and 60.2 °C, respectively (Figure 7B). With respect to **O_2_N-BZM7** and **O_2_N-BZM9**, the *T*_m_ values determined were 57.5 °C and 58.4 °C (Figure 7B). The TPI enzyme was most affected in the presence of **O_2_N-BZM7**, with a loss of 3 °C in thermal stability (*T*_m_ value of 57.5 °C), while the rest of the compounds did not show significant changes in their *T*_m_ values. These data confirm that **O_2_N-BZM7** has a strong effect on the global stability of the TPI enzyme from *G. lamblia*. However, it is interesting to note that, although the compounds caused alterations in catalysis and a loss in the secondary structure of the TPI protein, they did not affect the overall stability of the protein; *T*_m_ values close to that of TPI free of treatment were determined. We suggest that no effect was observed on the thermal stability of the TPI enzyme because the compounds were synthesized to react with the Cys of the TPI enzyme; this reaction is local and would not disrupt the global stability of the protein.

#### 2.6.3. Intrinsic and Extrinsic Fluorescence Assays

**Figure 8:**
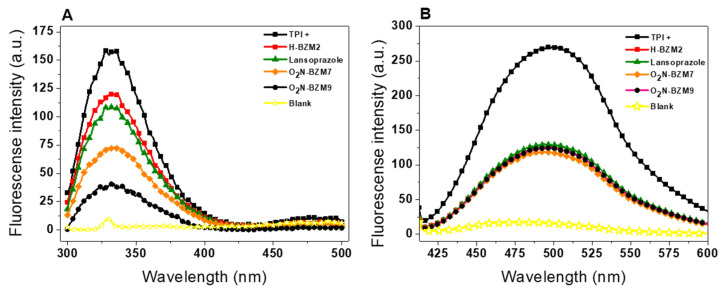
Fluorescence emission spectra of the TPI enzyme from *G. lamblia*. (**A**) Intrinsic fluorescence spectra of the TPI enzyme, and (**B**) 8-Anilinonaphthalene-1-sulphonate (ANS) assays in the absence or presence of the four synthesized compounds incubated at the IC_50_ concentration. Values obtained from buffer containing ANS without protein (open stars) were subtracted from the recordings with protein. The experimental conditions for all the experiments are described in Section 3.6.1 of the Materials and Methods. The data are the mean of at least four independent experiments.

Previously, it has been reported that TPI from *G. lamblia* was affected at the functional and structural level by the use of sulfhydryl reagents such as 5,5′-Dithiobis-2-Nitrobenzoic Acid; DNTB) and 2-carboxyethyl methanethiosulfonate (MTSCE) [13]. Furthermore, Hernández-Ochoa et al. [17] reported that the derivatization of Cys residues of TPI from *G. lamblia* induces a reduction in the maximum peak fluorescence when using the two novel proton pump inhibitor (PPI) derivatives **BHO2** and **BHO3**. Based on the above, we performed intrinsic and extrinsic fluorescence assays to determine the changes in the tertiary structure of TPI protein. The intrinsic fluorescence of the four tryptophan residues contained in the TPI/monomer was monitored to determine structural changes in the presence of the four synthesized compounds. We found that the intrinsic fluorescence of the native TPI protein without inhibitors showed a peak at 330 nm, with a maximum intensity of 161 arbitrary units (a.u.; Figure 8A), while in presence of the compounds there was a reduction in the maximum peak of fluorescence of 25–75% of the initial signal intensity (Figure 8A). In the presence of **H-BZM2** (40.1 a.u.) and lansoprazole (123 a.u.), the maximum intensity of fluorescence of the TPI enzyme decreased by 75% and 25%, respectively (Figure 8A), while in the presence of **O_2_N-BZM7** and **O_2_N-BZM9**, the maximal fluorescence intensity was 107 and 72.6 a.u, respectively, indicating a reduction of 33% and 55%, respectively, compared to the TPI enzyme free of compounds. It is interesting to note that the four synthesized compounds showed a higher reduction in the maximum fluorescence intensity of the TPI enzyme with respect to the PPI derivative **BHO2**, where 25% of the signal was lost. As previously noted, the decrease in fluorescence intensity could be due to the fact that the four tryptophan residues were exposed to the environment after incubation with the compounds and induced unfolding of the native three-dimensional (3D) structure in the presence of the four compounds, which had a negative effect on the activity of the TPI enzyme. Furthermore, these results are correlated with the loss of catalytic activity and are in concordance with the biochemical assays, in which the compounds and lansoprazole were faster to form the enzyme‒inhibitor complex, showed alterations on the secondary and 3D structure of the TPI protein, and had an inhibitory effect on the viability of *G. lamblia*.

Another probe to identify alterations in the 3D structure of proteins is the extrinsic fluorescent molecular probe by ANS assay, which is a molecule that has a high affinity for hydrophobic regions and exhibits higher ANS fluorescence signals. Based on the above, we measured the fluorescence emission spectra of ANS in the presence of the four synthesized compounds. As seen in Figure 8B, all the compounds induced alterations in the 3D structure of the TPI protein, with the extrinsic fluorescence intensities in the presence of the four inhibitors decreasing with respect to the enzyme without the inhibitor. The results obtained indicate that the TPI enzyme with ANS used as a control (without compounds) had a maximal fluorescence emission spectrum of 275 a.u. (corresponding to 100% fluorescence intensity) at 485 nm (Figure 8B), while the TPI enzyme incubated with the compounds produced an ANS spectrum with maximal fluorescence at 125 nm, 2.2-fold less than with 100% fluorescence intensity; this behavior was observed for all the compounds. Thus, the decrease in fluorescence intensity by more than 100% in the derivatized TPI enzyme is clear evidence that the enzyme does not expose buried hydrophobic regions after treatment with the compounds. These results are in agreement with what was obtained with the TIM from the human parasite *Encephalitozoon intestinalis* (EiTIM), in which a decrease in the fluorescence intensity was also observed [27].

Finally, it is interesting to note that all the results showed in this work indicate that the TPI of *Giardia lamblia* caused alterations in the enzymatic activity, secondary structure, global stability, and tertiary structure of the protein when it was incubated with the synthesized compounds. The compounds had a good inhibitory effect on the viability of *G. lamblia*, and no effect on the viability of human intestinal cells, making them good candidates to be proposed as antigiardial drugs.

### 2.7. Molecular Docking Studies

**Figure 9:**
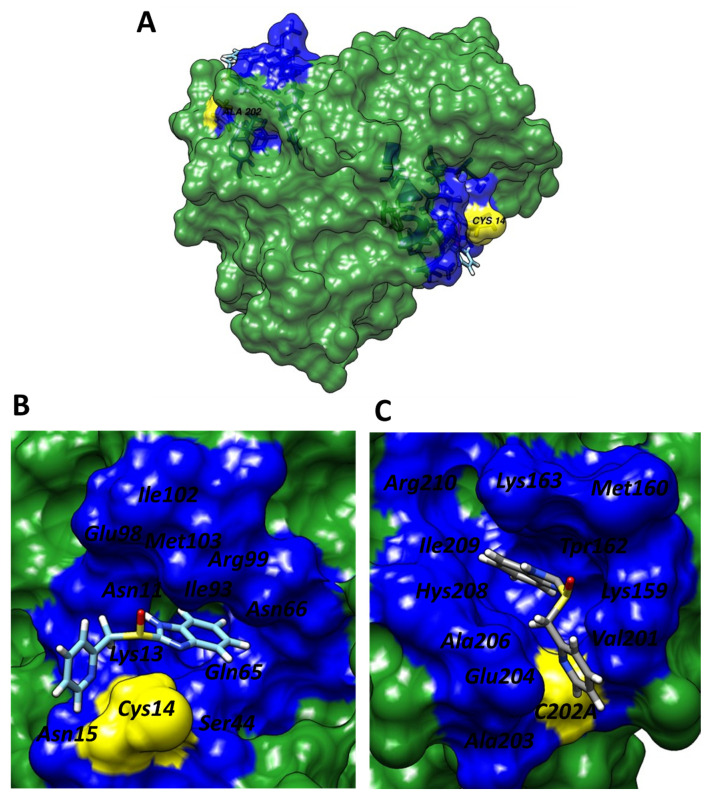
Docking prediction between TPI protein (PDB entry 4BI7) from *Giardia lamblia* with the compounds. (**A**) A general view of the binding affinities of all the antigiardial compounds with the TPI from *G. lamblia*. (**B**) First zone of interaction of all the compounds with the amino acids common to the binding pocket (blue) near Cys14 (yellow). (**C**) Second zone of interaction of all the compounds in the TPI structure, showing amino acids common to the binding pocket (blue) near C202A (yellow).

To explore the binding affinities of H-BZM2, lansoprazole, O_2_N-BZM7, and O_2_N-BZM9 antigiardial compounds with the TPI from *G. lamblia* (PDB entry 4BI7), we performed a molecular blind docking analysis. In Figure 9, the in silico study revealed two main zones of interaction for all the compounds (Figure 9A). The first zone was found near the Cys14 residue; in this pocket, 12 amino acids were identified that interact with the compounds Asn11, Lys13, Cys14, Asn15, Ser44, Gln65, Asn66, Ile93, Glu98, Arg99, Ile102, and Met103 (the amino acid number corresponds to the TPI sequence from *G. lamblia*; Figure 9B); the second zone was near Cys202 residue. In the case of crystal 4B17, Cys202 is mutated by the amino acid alanine, and 12 amino acids were identified that interact with the compounds Lys159, Met160, Trp162, Lys163, Val201, Ala202, Ala203, Glu204, Ala206, His208, Ile209, and Arg210 (Figure 9B). It is interesting to note that all the interactions found for each of the compounds were different.

**Figure 10:**
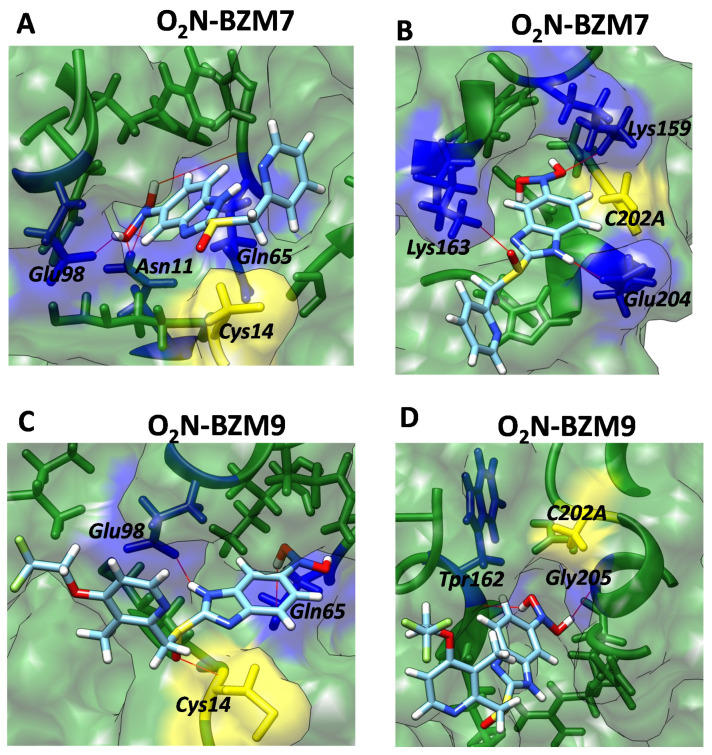
Prediction of docking between TPI protein (PDB entry 4BI7) and **O_2_N-BZM7** and **O_2_N-BZM9**: 3D representations of the binding model of **O_2_N-BZM7** in the vicinity of (**A**) Cys14 and (**B**) C202A, and of **O_2_N-BZM9** in the vicinity of (**C**) Cys14 and (**D**) C202A of TPI from *G. lamblia*. The interactions are shown in red, and Cys14 and C202A are shown in yellow.

The docking predicted with compounds O_2_N-BZM7 and O_2_N-BZM9 (compounds having a nitro group at Position 5 of the benzimidazole ring (R_1_)) showed an increase in the formation of hydrogen bridges with respect to the other compounds. We found that near Cys14, the nitro group of O_2_N-BZM7 forms four hydrogen bridges with the amino acids Asn11, Gln65, and Glu98 (Figure 10A); near Cys202, three hydrogen bridges are formed with amino acids Lys159, Lys 163, and Glu204 amino acids (Figure 10B). With respect to compound O_2_N-BZM9, we observed that near the Cys14 residue, this compound formed three hydrogen bonds with amino acids Cys14, Gln65, and Glu98 (Figure 10C). In addition to the predicted binding near Cys202, O_2_N-BZM9 showed interactions with Tpr162 and Gly205 (Figure 10D).

Previously, it was reported that the sulfur atom of the benzimidazole ring forms a covalent bond with the cysteine residues present in the TPI protein from *G. lamblia* [17], which leads to its loss of activity; the docking study revealed that the nitro group of compounds O_2_N-BZM7 and O_2_N-BZM9 possesses the ability to form up to four hydrogen bonds, which probably causes them to have a greater inhibitory effect on the TPI enzyme from *G. lamblia* with respect to H-BZM1, H-BZM2, H_2_N-BZM6, and lansoprazole, which do not have the nitro substituent. These results strongly suggest that the nitro group is essential in the benzimidazole ring (R_1_) to enhance the inhibitory activity of the compounds, which was confirmed by the values of *k*_2_ shown by O_2_N-BZM7 and O_2_N-BZM9 (3.2 M^−1^ s^−1^ and 2.8 M^−1^ s^−1^, respectively).

**Figure 11:**
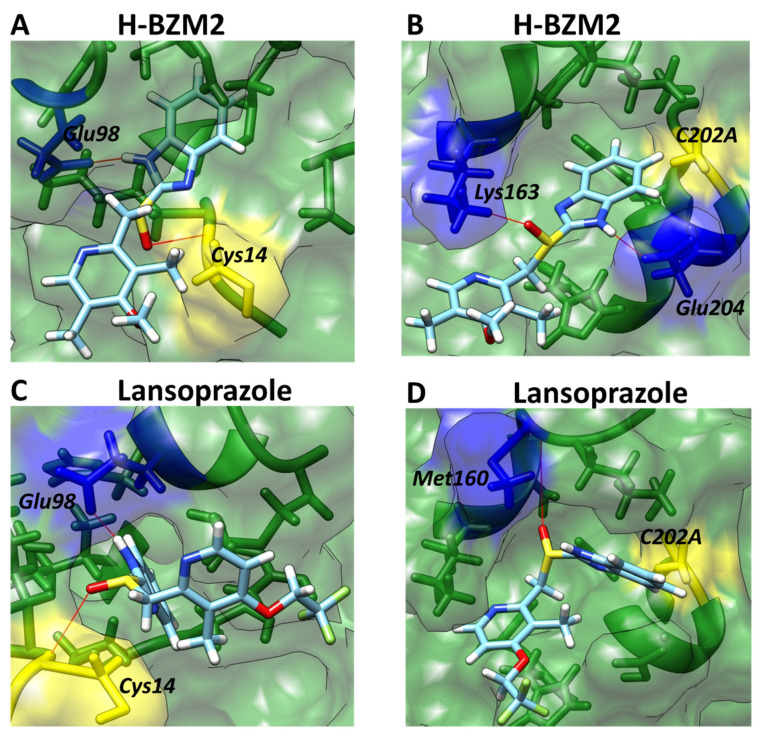
Prediction of docking between TPI protein (PDB entry 4BI7) and **H-BZM2** and lansoprazole: 3D representations of the binding model of **H-BZM2** in the vicinity of (**A**) Cys14 and (**B**) C202A, and of lansoprazole in the vicinity of (**C**) Cys14 and (**D**) C202A of TPI from *G. lamblia*. The interactions are shown in red, and Cys14 and C202A are shown in yellow.

Regarding H-BZM2, which experimentally showed a *k*_2_ value of 2.3 M^−1^ s^−1^, the docking analysis showed that two interactions are formed between the benzimidazole ring and the amino acids Glu98 and Cys14 (Figure 11A). On the other hand, in the pocket of C202A, the benzimidazole ring forms two interactions with the amino acids Lys163 and Glu204 (Figure 11B). Finally, for lansoprazole, the docking analysis showed that two interactions are formed between the benzimidazole ring and the amino acids Cys14 and Glu98 (Figure 11C); in the pocket of C202A, the benzimidazole ring forms only one interaction with the amino acids Met160 (Figure 11D), and this compound showed a *K*_2_ value of 1.78 M^−1^ s^−1^.

## 3. Materials and Methods

### 3.1. Chemicals

All chemicals used in the synthesis of compounds were purchased from Sigma-Aldrich (St. Louis, MO, USA) were used as received without any prior purification. Penicillin/streptomycin (5000 U/mL), Trypsin-EDTA (0.25%), phenol red, and Eagle’s Minimum Essential Medium (EMEM) were purchased from Gibco, Life Technologies (Paisley, UK). The Caco-2 (ATCC HTB-37; Manassas, VA, USA) and HT-29 (ATCC HHTB-38) cell lines were purchased from Sigma-Aldrich (Castle Hill, NSW, Australia). Chemicals used for the in vitro culture of *G. lamblia* were sourced as follows: glucose and L-cysteine (ACROS organics, Thermo Fisher Scientific, Scoresby, VIC, Australia), ammonium iron (III) citrate, ascorbic acid (Sigma-Aldrich, Castle Hill, NSW, Australia), potassium dihydrogen orthophosphate (UNIVAR, Ingleburn, NSW, Australia), bovine bile (Fluka analytical (BD)), and di-potassium hydrogen orthophosphate (Fronine Laboratories and Supplies, Riverstone, NSW, Australia).

### 3.2. Chemistry

Melting points were determined in an A9200 digital melting point instrument from Thermo Scientific; the results are uncorrected. All reactions were monitored by thin-layer chromatography (TLC) and carried out using glass plates precoated with silica gel 60 F254 (Merck, Kenilworth, NJ, USA). ^1^H NMR spectra were determined on Varian 600 MHz AR Premium Compact and ^13^C NMR (150 MHz) instruments (Varian-Agilent, Santa Clara, CA, USA). Chemical shifts are reported in ppm DMSO-d6 and CDCl_3_ as deuterated solvents. Mass spectrometry was obtained from a JEOL JMS-700 spectrometer by electronic impact (JEOL, Tokyo, Japan).

### 3.3. General Procedure for the Synthesis of Compounds

Five compounds were designed on the basis of the proton pump inhibitor omeprazole and lansoprazole, maintaining the 2-(2-pyridylmethylsulfinyl)-1*H*benzimidazole core. **H-BZM1**, **H-BZM2**, and lansoprazole have a hydrogen at Position 5 of the benzimidazole ring (R_1_); with respect to the pyridine ring **H-BZM1** has no substituents, **H-BZM2** and **O_2_N-BZM7** have the same as omeprazole, **H_2_N-BZM6** and **O_2_N-BZM9** has the same as lansoprazole. Compounds were synthesized in the Laboratory of Immunochemistry, Hospital Infantil de México Federico Gómez (Ciudad de Mexico, Mexico). The synthesis of the derivatives was carried out according to the following general procedure, in two steps: first, the precursors were prepared in a mixture of 2-mercapto-5-benzimidazole (0.5 g, 3.04 mmoles) and the substituted 2-chloromethyl pyridine (0.55 g, 3.3 mmoles) in 10 mL of 1,2-dimethoxyethane (GLIMA), potassium hydroxide (0.34 g, 6 mmoles) was added, and the mixture was stirred for 8 h at 50 °C to give the desired 2-[(pyridin-2-ylmethyl)sulfanyl]-1H-benzimidazole compound. The total conversion of primary reagents to 2-[(pyridin-2-ylmethyl)sulfanyl]-1H-benzimidazole was monitored by TLC assay. Later water was added and the reaction mixture was extracted with CHCl_3_ (2 × 20 mL). The organic layer was dried over anhydrous Na_2_SO_4_ and concentrated under reduced pressure, and the residue was purified using column chromatography on silica gel, with CH_2_Cl_2_‒hexane (3:1 *v*/*v*) as the eluent, to give a pale yellow solid.

Second, smooth oxidation of the thioether group in precursors was carried out to obtain the final sulfoxide compounds. The oxidation was done according to the following general procedure: to a stirring mixture of pyridin-sulfanyl benzimidazole (0.2 g, 0.78 mmoles) in 5 mL of CHCl_3_ at 0 °C was added 3-chloroperoxybenzoic acid (0.19 g, 1.12 mmoles in 7 mL of CHCl_3_); the peracid was added slowly, drop by drop, and the reaction was monitored every 5 min by TLC in order to prevent sulfone formation. After completion of the reaction, the mixture was treated with a saturated solution of sodium bicarbonate (20 mL) and extracted with CHCl_3_ (2 × 20 mL). The organic layer was dried over anhydrous Na_2_SO_4_ and the solvent was removed under reduced pressure. The residue was purified by means of column chromatography on silica gel using CH_2_Cl_2_‒hexane (3:1 *v*/*v*) as the eluent to produce the desired product as a pale yellow solid, and their chemical structures were confirmed by NMR spectroscopy. The analogs labeled **H-BZM1**, **H-BZM2**, **H_2_N-BZM6**, **O_2_N-BZM7**, **O_2_N-BZM9** and the lansoprazole were stored at 4 °C until use. Stock solutions of analogs were prepared by dissolving them in dimethyl sulfoxide (DMSO).

*2-{[(pyridin-2-yl)methyl]sulfanyl}-1H-benzimidazole* (**H-BZM1**), light-yellow solid. Yield 85%, mp. 121.3–122.9 °C. ^1^H NMR (400 MHz, DMSO-d6): δ 4.52–5.00 (2H, *J* = 11.5 Hz CH2OS), 7.03–7.11 (1H, dd, *J* = 7.4, 1.2 Hz, H-6), 7.12–7.13 (1H, dd, *J* = 8.1, 1.7 Hz, H-3′), 7.24 (1H, ddd, *J* = 8.0, 6.9, 1.3 Hz, H-5′), 7.45 (ddd, *J* = 7.6, 1.2, 0.5 Hz, H-4)), 8.42 (1H, ddd, *J* = 5.1, 1.9, 0.5 Hz, H-6′) ppm. 13C NMR (175 MHz, DMSO-d6) δ: 21.3 (CH3); 61.9 (CH2); 123.2 (C-5′); 124.8 (broad, C-6), 125.3 (C-3′); 132.8 (broad, C-5); 136.9 (C-4′); 149.7 (C-6′); 150.7 (C-2′); 153.2 (C-2) ppm. Carbons C3a, C7a, C4 and C7 were not observed due to tautomeric equilibrium. MS calculated for C_13_H_11_N_3_OS: 257.31, found 255.

*2-[(4-methoxy-3,5-dimethylpyridin-2-yl)methanesulfinyl]-1H-benzimidazole* (**H-BZM2**), light-brown solid. Yield 72%, mp. 221.3–222.9 °C. ^1^H NMR (400 MHz, DMSO-d6). δ 2.07 (3H, s, CH3), 2.14 (3H, s, CH3), 3.54 (3H, s, CH3O), 4.63–4.71 (2H, *J* = 13.5 Hz, CH2), 7.19 (1H, dd, *J* = 8.0, 1.3 Hz, H-6), 7.22 (1H, dd, *J* = 8.1, 1.7 Hz, H-4)), 7.24 (1H, dd, *J* = 8.1, 0.5 Hz, H-7), 8.13 (1H, s, H-6′) ppm. ^13^C NMR (100 MHz, CDCl3) δ: 11.64 (CH3), 13.48 (CH3), 21.87 (CH3), 60.01(CH3O), 60.85 (CH2SO), 126.54 (C-5′), 127.15 (C-3′), 148.7 (C-2′), 149.83 (C-6′), 164.5 (C-4′); too broad resonances only detected through HMBC: 111.96 (C-4), 119.99 (C-7), 125.09 (C-6), 126.27, 133.1, 134.7, 142.18, 152.5 (C-2) ppm. MS calculated for C_17_H_19_N_3_O_2_S: 329.1198, found: 328.

*2-{[3-methyl-4-(2,2,2-trifluoroethoxy)pyridin-2-yl]methanesulfinyl}-1H-benzimidazole* (lansoprazole), brown solid. Yield 75%. Light-yellow solid. Mp. 121.3–122.9 °C. The results spectroscopic were accordingly with those previously published [25]. MS calculated for C_16_H_14_F_3_N_3_O_3_S: 369.36, found 367.

*2-{[3-methyl-4-(2,2,3-trifluoropropoxy)pyridin-2-yl]methanesulfinyl}-1H-benzimidazol-5-amine* (**H_2_N-BZM6**), light-yellow solid. Yield 70%. Mp. 121.3–122.9 °C. ^1^H NMR (400 MHz, DMSO-d6) δ: 2.27 (3H, s, CH3), 4.36–4.38 (2H, *J* = 13.6 Hz, CH2-SO), 4.40 (2H, q, *J* = 8.7 Hz, CH2-CF3), 6.57–6.59 (1H, d, *J* = 4.5 Hz, H-5′, Py), 6.67–6.69 (1H, dd, *J* = 8.3, 0.6 Hz, H-6), 6.79 (1H, dd, *J* = 1.8, 0.6 Hz, H-4), 7.29–7.32 (1H, d, *J* = 4.5 Hz, H-7), 8.35. (1H, d, *J* = 5.7 Hz, H-6′, Py) ppm. 13C NMR (100 MHz, CDCl3) δ: 10.77 (CH3), 34.91 (CH2CF3), 64.91 (CH2SO), 65.57 (CH2O), 106.01 (C-5′), 111.93 (C-3′), 111.7 (C-4), 121.08 (C-7), 121.74 (C-6), 124.32, 141.99, 147.53 (C-6′), 157.68 (C-2′), 162.50 (C-4′), ppm. MS calculated for C_17_H_17_F_3_N_4_O_2_S: 398.40, found 396.

*5-nitro-2-[(pyridin-2-yl)methanesulfinyl]-1H-benzimidazole* (**O_2_N-BZM7**), light-yellow solid. Yield 85%. Mp. 121.3–122.9 °C. The results spectroscopic were accordingly with those previously published [23]. MS calculated for C_13_H_10_N_4_O_3_S: 302.30, found 300.

*2-{[3-methyl-4-(2,2,2-trifluoroethoxy)pyridin-2-yl]methanesulfinyl}-5-nitro-1H-benzimidazole* (**O_2_N-BZM9**), light-yellow solid. Yield 70%. Mp. 121.3–122.9 °C. ^1^H NMR (400 MHz, DMSO-d6) δ: 2.23 (3Hs, CH3), 4.37, 4.39 (2H, *J* = 13.6 Hz, CH2-CF3), 4.72, 4.75 (2H, *J* = 13.6 Hz, CH3SO), 6.71 (1H, d, *J* = 4.4 Hz, H-5′, Py), 7.38 (1H, dd, *J* = 8.8, 0.5 Hz, H-6), 7.51 (1H, broad, H-4), 8.22 (1H, d, *J* = 5.7 Hz, H-6′, Py), 8.34 (1H, dd, *J* = 2.0, 0.5 Hz) ppm. 13C NMR (175 MHz, DMSO-d6) δ: 21.3 (CH3); 61.9 (CH2); 123.2 (C-5′); 124.8 (broad, C-6), 125.3 (C-3′); 132.8 (broad, C-5); 136.9 (C-4′); 149.7 (C-6′); 150.7 (C-2′); 153.2 (C-2) ppm. Carbons C3a, C7a, C4 and C7 were not observed due to tautomeric equilibrium. MS calculated for C_16_H_13_F_3_N_4_O_4_S: 414.35, found 412.

### 3.4. In Vitro Assays

#### 3.4.1. Antigiardial Activity

*Giardia lamblia* WB strain (ATCC number 30957) was grown in a modified TYI-S-33 medium supplemented with 10% fetal bovine serum. The parasite was cultivated in 15 mL screw-capped borosilicate glass tubes containing 13 mL medium. The tubes were incubated on a 15° horizontal slant at 37 °C. *Giardia* was harvested from confluent culture by chilling the tubes on ice for 5‒10 min to detach cells, followed by centrifugation at 800× *g* for 5 min.

The antigiardial activity of the prepared compounds was tested as described. One milligram of a test compound was dissolved in 10 L of DMSO and topped up with 1 mL growth medium. The solution was filter-sterilized using 0.22-m syringe filters and the appropriate volumes of the solutions were taken to prepare the concentration of each compound in 1.5 mL microtubes. For each preparation, concentrations of 500, 250, 125, 62, and 31 g/mL were prepared in a final volume of 1.5 mL. Each tube was inoculated with 5 × 10^4^ trophozoites of *G. lamblia* and then incubated for 48 h at 37 °C. After incubation, 75 L of the treated trophozoites were subcultured individually for another 48 h in a fresh medium free of compounds. At the end of this period, the viable trophozoites were counted. Each compound was assayed in duplicate in three independent experiments. In each assay, appropriate controls were also tested, including a sample without compound and another with DMSO.

The parasites in each tube were counted using a standard hemocytometer at 10× objective. In each count, trypan blue was employed to distinguish live parasites from dead ones. To permit detachment of *Giardia*, the tubes were placed on ice for 10 min and the parasites were then centrifuged at 800× *g* for 10 min. The supernatant was discarded and 1 mL fresh medium was added to each tube. The final suspension was prepared by mixing 25 μL of the parasite suspension in each tube with 100 μL of 0.4% trypan blue in phosphate-buffered saline (PBS). The 50% inhibitory concentration (IC_50_) was employed as a parameter for biological activity. The IC_50_ is the concentration of compounds that cuts the number of parasites to half that in the negative control (growth medium + DMSO + parasites). Lastly, the 50% inhibitory concentration (IC_50_) was calculated.

#### 3.4.2. In Vitro Cytotoxicity

The cytotoxicity of compounds was investigated on human cell lines Caco-2 (ATCC HTB-37) and HT-29 (ATCC HHTB-38), using a Cell Proliferation Kit II (XTT; St Louis, MO, USA) according to the manufacturer’s instructions. A cell suspension of 10^5^ cells/mL was prepared in Dulbecco’s modified Eagle’s (DMEM); medium supplemented with 10% inactivated fetal bovine serum and 1% Penicillin/streptomycin (5000 U/mL), from confluent cultures, and 100 mL portions of the suspension were added to the wells of 96-well plates. The cells were incubated for 24 h at 37 °C in an atmosphere of 5% CO_2_, and the medium in each well was then replaced with 150 L fresh medium. Solutions of the compound were prepared and sterilized as described in Section 3.4.1 of Materials and Methods. Then, 150 μL of two-fold serial dilutions of each of the compounds, starting at a concentration of 500 M, in a culture medium at a concentration of 0.3% DMSO, were prepared in the plates. After 48 h of incubation at 37 °C in an atmosphere of 5% CO_2_, the viability of cells in each well was determined as follows: The medium in each well was gently replaced with 100 L of fresh medium, 50 µL of XTT labeling mixture were added per well, and the mixture was incubated for 4 h at 37 °C in an atmosphere of 5% CO_2_ and the absorbance at 550 nm measured. Each compound was assayed in triplicate via three independent experiments. The CCI_50_ values were determined using the nonlinear regression function of GraphPad Prism version 6.04 for Windows, GraphPad Software, La Jolla, CA, USA, https://www.graphpad.com (accessed on 23 February 2020).

### 3.5. In Vitro Screening of TPI Inactivation

#### 3.5.1. Purification of TPI Recombinant Enzyme

The TPI from *G. lamblia* was expressed and purified [13]. Briefly, bacteria containing the plasmid for TPI was grown in LB medium supplemented with 0.1 mg/mL of ampicillin and incubated at 37 °C. When the culture reached A_600_ = 0.8, it was induced using 0.4 mM IPTG and incubated overnight at 30 °C with shaking at 180 rpm. After the bacteria were collected by centrifugation (10,000× *g*, 15 min) and suspended in 40 mL of lysis buffer, pH 8.0 (50 mM Tris, 50 mM NaCl, 5 mM β-mercaptoethanol, and 1 mM phenylmethylsulfonyl fluoride). The bacterial suspension was disrupted by sonication and centrifuged at 9000× *g* for 1 h at 4 °C. Protein purification was performed by immobilized metal affinity chromatography (IMAC) using a Profinity Ni^2+^ charged resin (Bio-Rad) previously reported by Enriquez-Flores et al. [13]. The His-tag sequence was removed from the TIM by incubation with TEVP at a molar ratio of 30:1 (TPI/TEVP) at room temperature for 17 h in digestion buffer, pH 8.0 (50 mM Tris, 0.5 mM ethylene diamine tetraacetic acid (EDTA), and 1 mM dithiothreitol). The enzyme concentration was spectrophotometrically determined at 280 nm, and the enzyme was used immediately.

#### 3.5.2. Enzyme Activity Assay

Enzyme activity of TPI was determined spectrophotometrically as described [17] by monitoring the conversion of glyceraldehyde-3-phosphate (GAP) to dihydroxyacetone phosphate (DHAP). Briefly, the conversion of GAP to DHAP using a coupling system with enzyme α-glycerol-3-phosphate dehydrogenase (α-GDH) was determinate by measuring NADH oxidation at 340 nm, at 25 °C. The standard reaction mixture contained 1 mM GAP, 0.2 mM NADH, and 0.9 units/mL of α-GDH in TE buffer (100 mM triethanolamine, 10 mM EDTA, pH 7.4). The reaction was initiated by adding 5 ng/mL of TPI to the reaction mixture.

#### 3.5.3. Inactivation of TPI with Compounds

To determine the inactivation of the TPI enzyme from *G. lamblia*, we performed inactivation assays of the enzyme in the presence of the synthesized compounds. The compounds’ stock solutions were freshly prepared using 100% dimethyl sulfoxide (DMSO) as the solvent at 25 mM. The inactivation assays were performed by incubating 0.2 mg/mL of the TPI enzyme for 2 h at 37 °C in TE buffer with increasing concentrations of the compounds (0 to 500 μM). DMSO was maintained at 5% during incubation; this concentration did not affect the enzyme activity. After the incubation period, the enzymatic activity was measured by withdrawing an aliquot of each experimental condition, as described above. The results were expressed in terms of the percentage of activity versus the compound’s concentration.

The second-order rate constants of inactivation were calculated by obtaining the pseudo-first-order rate constant (*k*_1_) values at each fixed concentration by fitting them to an exponential decay curve. Then, the *k*_1_ values were replotted against the concentrations of each compound; the second-order rate constant value of inactivation was obtained from the slope of these plots.

### 3.6. Spectroscopic Characterization

#### 3.6.1. Circular Dichroism (CD) and Thermal Stability Assays

All spectroscopic assays were carried out at a protein concentration of 0.2 mg/mL. Changes in the secondary structure of proteins were monitored by circular dichroism (CD) in a Jasco-810 spectropolarimeter containing a thermostated Peltier-controlled cell holder. For CD experiments, the enzyme was suspended in 50 mM phosphate buffer (buffer P), pH 7.4, and incubated with the four synthesized compounds by 2 h at 37 °C. Spectral scans from 190 to 280 nm at 0.1 nm intervals were performed at 25 °C using a quartz cell with 0.1 cm path length. All CD data were reported as molar ellipticity. In addition, the thermostability of modified TPI enzyme in the absence or presence of each of the synthesized compounds was evaluated, recording the loss of CD signal at 222 nm during a temperature scan ranging from 25 to 75 °C and an increase of 1 °C/2.5 min. The unfolded fraction of enzyme and melting temperature (*T*_m_) was determined as reported before. The experiments were performed in triplicate.

#### 3.6.2. Intrinsic and Extrinsic Fluorescence Assays

Changes in tertiary structure were analyzed following the intrinsic and extrinsic fluorescence of the TPI enzyme in a Perkin-Elmer LS-55 spectrofluorometer (Perkin-Elmer, Wellesley, MA, USA). The TPI protein was adjusted at 0.1 mg/mL in 50 mM phosphate buffer (buffer P), pH 7.4n, and incubated with the four synthesized compounds by 2 h at 37 °C. After the TPI protein was incubated with the synthesized compounds, the samples were excited at 280 nm and we recorded the emission spectra from 300 to 500 nm with a scan speed of 150 nm/min and using excitation and emission slits of 5 and 4 nm, respectively. Furthermore, we determined the extrinsic fluorescence of the TPI enzyme in the presence of the four compounds by 8-anilinonaphthalene-1-sulfonic acid (ANS) assay to monitor changes in the hydrophobic regions of the protein. The protein was incubated in the same conditions as the intrinsic fluorescence assay. Then, we recorded the fluorescence spectra from 400 to 600 nm in the presence of 25 mM of ANS dissolved in methanol at 25 °C with a scanning rate of 150 nm/min. The samples were excited to 395 nm using slits of excitation and emission of 10 nm, respectively. The final spectrum was the average of five scans. Later, each spectrum was subtracted from the spectra of the blank (buffer P solution containing each of the compounds plus ANS).

### 3.7. In Silico Analysis of the TPI Crystallographic Structure from G. lamblia

Here, we used the crystal structure of mutant (C202A) TPI from *Giardia lamblia*, complexed with 2-phosphoglycolic acid with access code PDB 4BI7 [28]. The atomic coordinates of TPI were submitted to the PDBsum server (PDBsum-EMBL-EBI) [29] to add the hydrogens to the structure. In order to identify all interactions on the TPI protein, blind docking was performed using the SwissDock Server (http://www.swissdock.ch/docking accessed on 23 June 2020) [30]. The ligand structures of **H-BZM2**, **lansoprazole**, **O_2_N-BZM7**, and **O_2_N-BZM9** were energy-minimized UCSF Chimera software developed by the Resource for Biocomputing, Visualization, and Informatics at the University of California, San Francisco, with support from NIH P41-GM103311 [31] and later docked on the crystal structure (PDB: 4B17). SwissDock generates all possible binding modes for each ligand; the most favorable binding modes at a given pocket are clustered. The predictions file provided Cluster Rank/Element Full Fitness and estimated binding free energy ΔG.

Chimera software was used to visualize the receptor–ligand interactions for all individual clusters obtained. For each compound, we analyzed the amino acids that interact with the ligand, the number of hydrogen bonds formed, the specific atoms involved, and the distance between them.

### 3.8. Statistical Analysis

The results of the in vitro drug efficacy studies were analyzed using GraphPad Prism. For the XTT (viability) assay, the mean and standard error of the mean were determined, with each assay completed in triplicate. The IC_50_ was calculated using the log (inhibitor) vs. normalized response—variable slope function in GraphPad Prism.

## 4. Conclusions

We reported five derivatives of 2-mercaptobenzimidazol named **H-BZM1**, **H-BZM2**, **H_2_N-BZM6, O_2_N-BZM7**, and **O_2_N-BZM9** with different substituent groups linked to the benzimidazole, which showed antigiardial activity, and without cytotoxic effects on mammalian HT29 and Caco-2 cells. The antigiardial activity of compounds was improved compared to the omeprazole and lansoprazole. The most active compounds were **H-BZM2, O_2_N-BZM7**, and **O_2_N-BZM9** with greater antigiardial activity (IC_50_: 36, 14, and 17 µM on trophozoites), and inhibited the TPI enzyme (*K*_2_: 2.3, 3.2 and 2.8 M^−1^ s^−1^) respectively. The plausible modes of action of compounds involve TPI inhibition, causing alterations in the enzymatic activity, secondary structure, global stability, and tertiary structure of the protein in the presence of the compounds. Finally, as suggested by docking study that the nitro group of compounds **O_2_N-BZM7** and **O_2_N-BZM9** (R_1_) possess the ability to form up to four hydrogen bonds, which probably causes them to have a greater inhibitory effect on the TPI enzyme from *G. lamblia.* The results presented in this study demonstrated that the class of benzimidazoles, related to PPIs, has the potential to be developed as antigiardial agents. The three analogs presented here were potent and fast-acting. For the above, the compounds **H-BZM2**, **O_2_N-BZM7**, and **O_2_N-BZM9** are an attractive potential starting point for new antigiardial drugs.

## Figures and Tables

**Table 1:** In vitro antigiardial activities of compounds, and the cytotoxic activity on Caco-2 and HT29 cells.

Compounds	IC_50_ (μM)	CC_50_ (μM)		IC_50_ (μM)
	*Giardia lamblia*	Caco-2 (SI)	HT29 (SI)	TPI
H-BZM1	676	4012 (6)	3918 (**5**)	>500
H-BZM2	36	3184 (88)	1912 (**53**)	37
Lansoprazole	45	2637 (59)	548 (**12**)	66
H_2_N-BZM6	135	4006 (29)	1888 (**14**)	>500
O_2_N-BZM7	14	640 (45)	519 (**37**)	12
O_2_N-BZM9	17	663 (39)	622 (**36**)	20
Omeprazole [17]	300	-	-	225

IC_50_: The concentration of compound that is required to kill 50% of trophozoites in vitro. CC_50_: Concentration of the compounds that killed 50% of the cells. The numbers inside the parentheses correspond to the ratio of CC_50_/IC_50_ values between mammal cells and trophozoites (selectivity index).

## Supplementary Materials

The following are available online, Figures S1, S2, S4, S5, S7, S8, S10, S11, S13, S14: Nuclear magnetic resonance spectra of compounds. Figures S3, S6, S9, S12, S15: High-resolution mass spectra of compounds.
