# Supplementary material for: Enhanced Antigiardial Effect of Omeprazole Analog Benzimidazole Compounds
Source: Molecules. 2020 Sep 1;25(17):3979. doi: 10.3390/molecules25173979 (PMC7504735; doi:10.3390/molecules25173979)
Supplement: Supplementary file 1 [file molecules-25-03979-s001.pdf]

SUPPLEMENTARY MATERIAL

Enhanced antigiardial effect of omeprazole analog benzimidazole compounds

Beatriz Hernández-Ochoa [1,*], Saúl Gómez-Manzo [2], Adrián Sánchez-Carrillo [1], Jaime Marcial-Quino [3], Luz María Rocha-Ramírez [4], Araceli Santos-Segura [1], Edson Jiovany Ramírez-Nava [2], Roberto Arreguin-Espinoza [5], Miguel Cuevas-Cruz [5], Alfonso Méndez-Tenorio [6], and Ernesto Calderón-Jaimes [1,*]

[1] Laboratorio de Inmunoquímica, Hospital Infantil de México Federico Gómez, Secretaría de Salud, Ciudad de Mexico 06720, Mexico; ausbir@yahoo.com.mx (A.S.-C); chelyss68@yahoo.com.mx (A.S.-S)

[2] Laboratorio de Bioquímica Genética, Instituto Nacional de Pediatría, Secretaría de Salud, Ciudad de México 04530, Mexico; saulmanzo@ciencias.unam.mx (S.G.-M); edsonjiovany@ciencias.unam.mx (E.J.R.-N)

[3] Consejo Nacional de Ciencia y Tecnología (CONACYT), Instituto Nacional de Pediatría, Secretaría de Salud, Ciudad de Mexico 04530, Mexico; jmarcialq@ciencias.unam.mx

[4] Departamento de Infectología, Hospital Infantil de México Federico Gómez, Dr. Márquez No. 162, Col Doctores, Delegación Cuauhtémoc 06720, Mexico; luzmrr7@yahoo.com.mx

[5] Departamento de Química de Biomacromoléculas, Instituto de Química, Universidad Nacional Autónoma de Mexico, Ciudad de Mexico 04510, Mexico; arrespin@unam.mx (R.A.-E.); miguel.ccqi@yahoo.com.mx (M.C.-C.)

[6] Laboratorio de Biotecnología y Bioinformática Genómica, Escuela Nacional de Ciencias Biológicas, Instituto Politécnico Nacional, Ciudad de México 11340, Mexico, amtenorio2000@gmail.com

* Correspondence: beatrizhb_16@comunidad.unam.mx (B.H.-O); ecalderj5@yahoo.com.mx (E.C.-J).

# Nuclear Magnetic Resonance Spectra of derivatives.

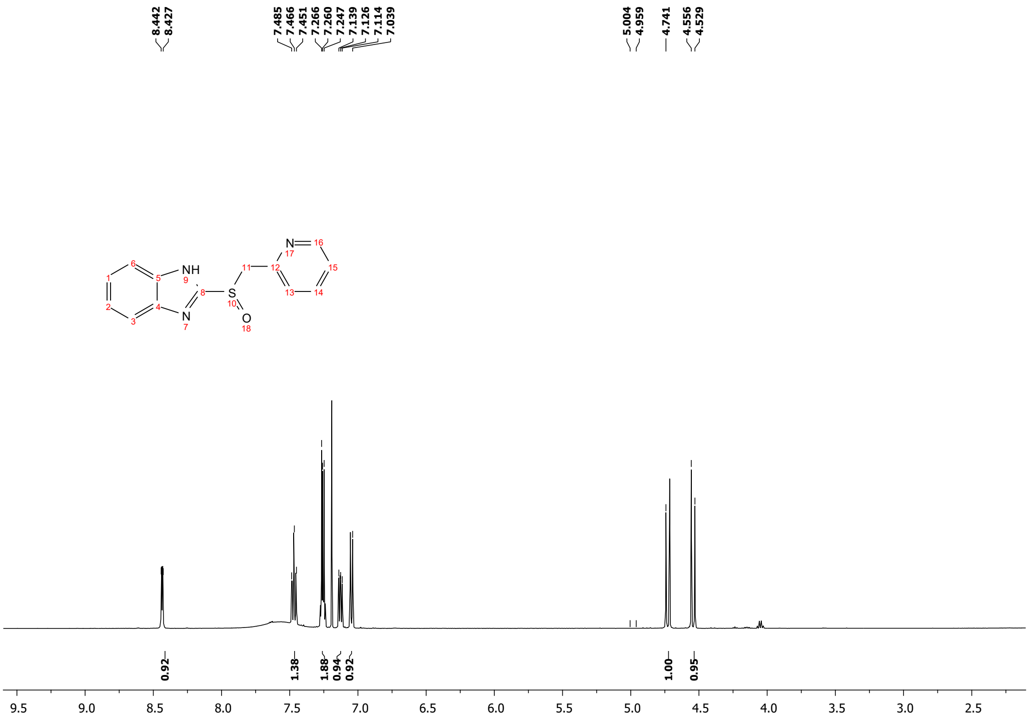

Figure S1. ^{1}H NMR spectrum of compound **H-BZM1** in CHCl3-*d6*.

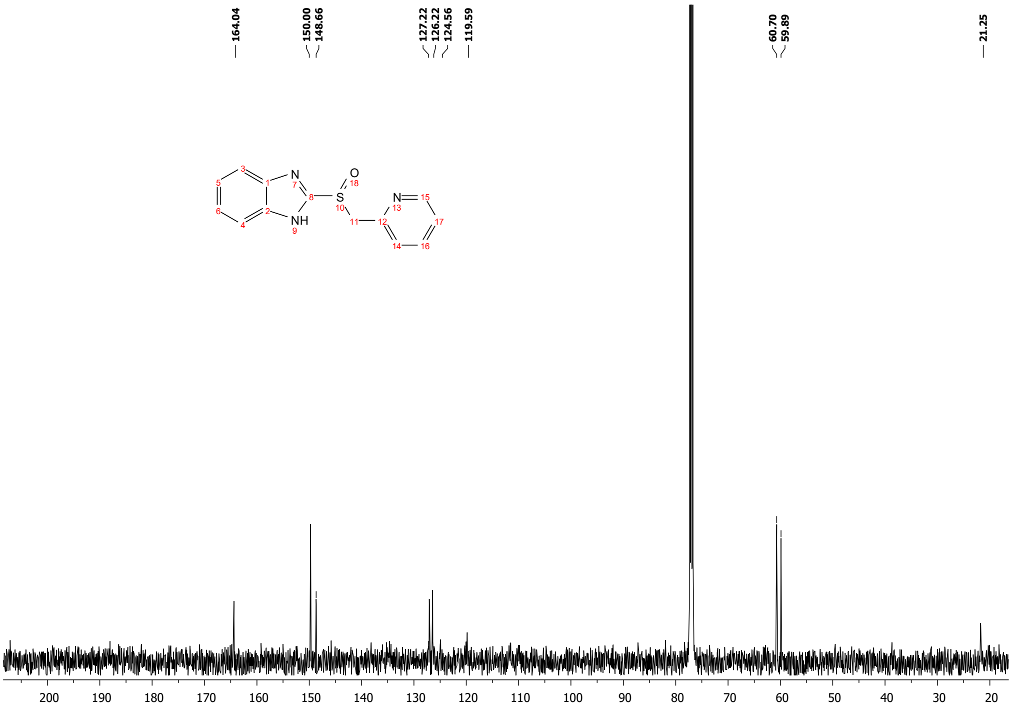

Figure S2. ^{13}C NMR spectrum of compound **H-BZM1** in CHCl3-*d6*.

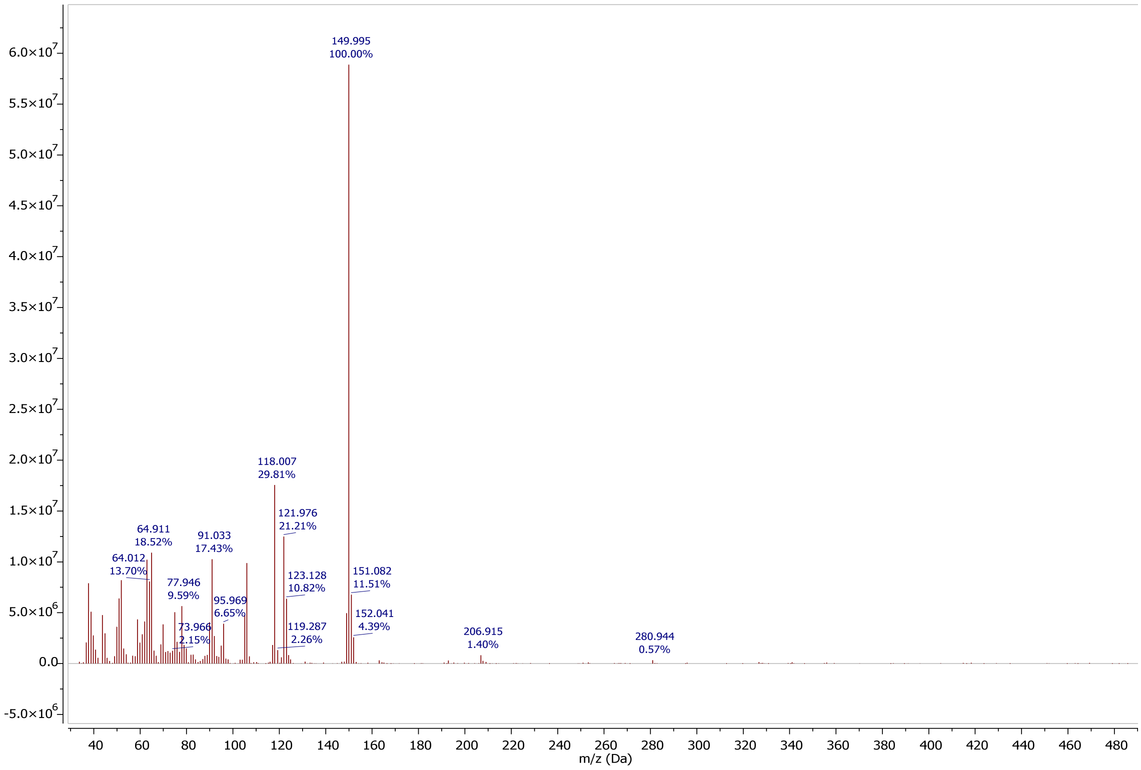

Figure S3. High-resolution mass spectra of compound **H-BZM1**

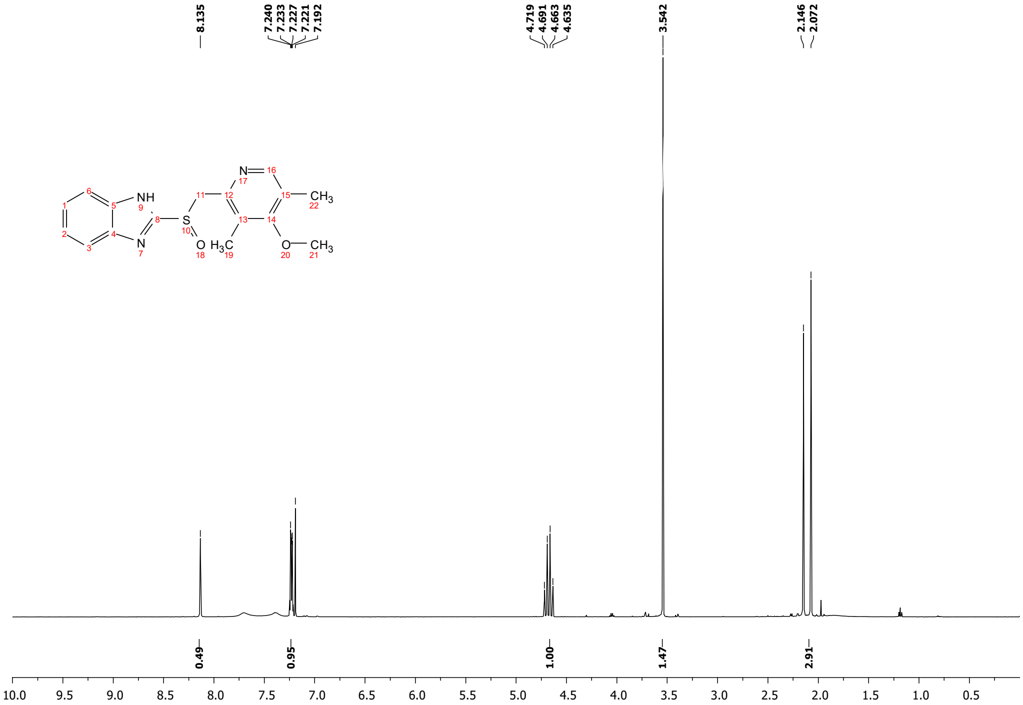

Figure S4. ^{1}H NMR spectrum of compound **H-BZM2** in $CHCl_3$-*d6*.

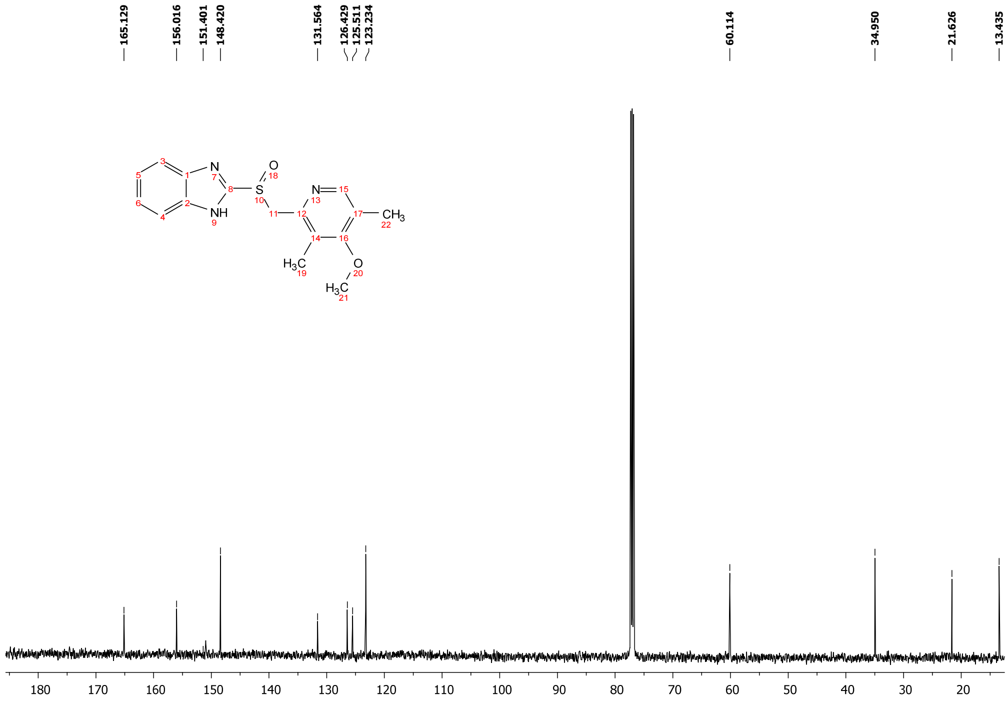

Figure S5. ^{13}C NMR spectrum of compound **H-BZM2** in $CHCl_3$-*d6*.

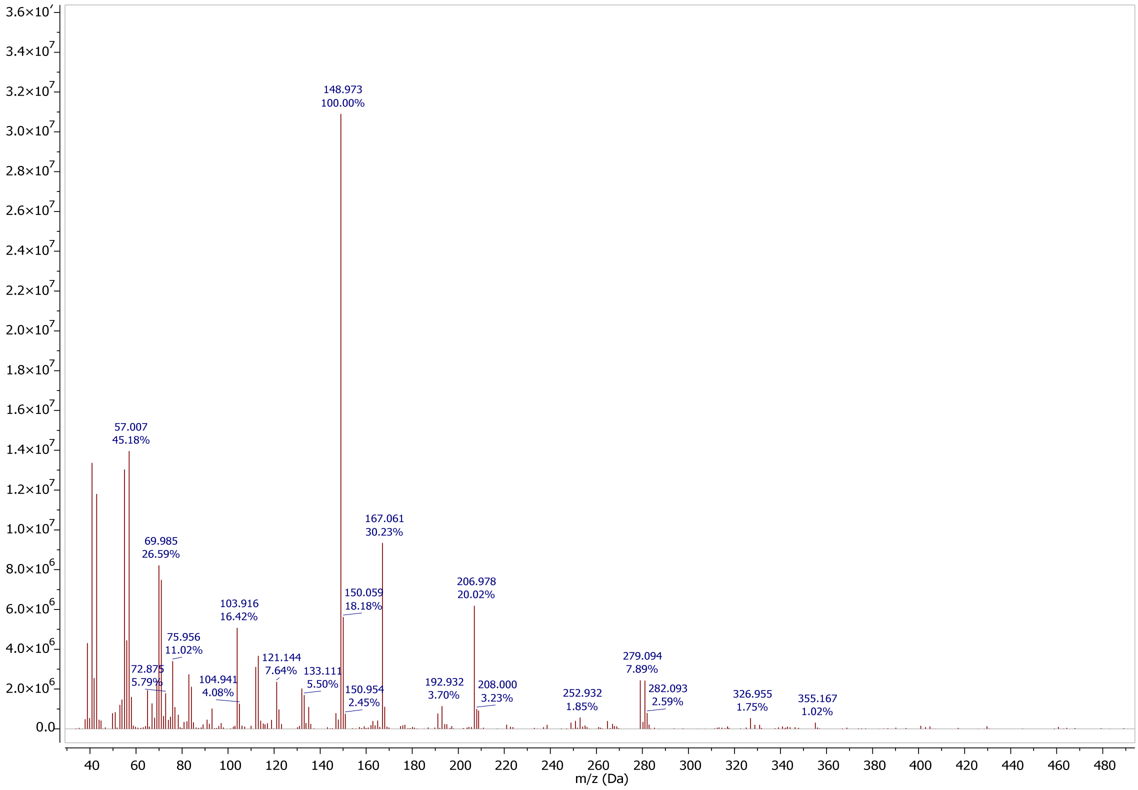

Figure S6. High-resolution mass spectra of compound **H-BZM2**

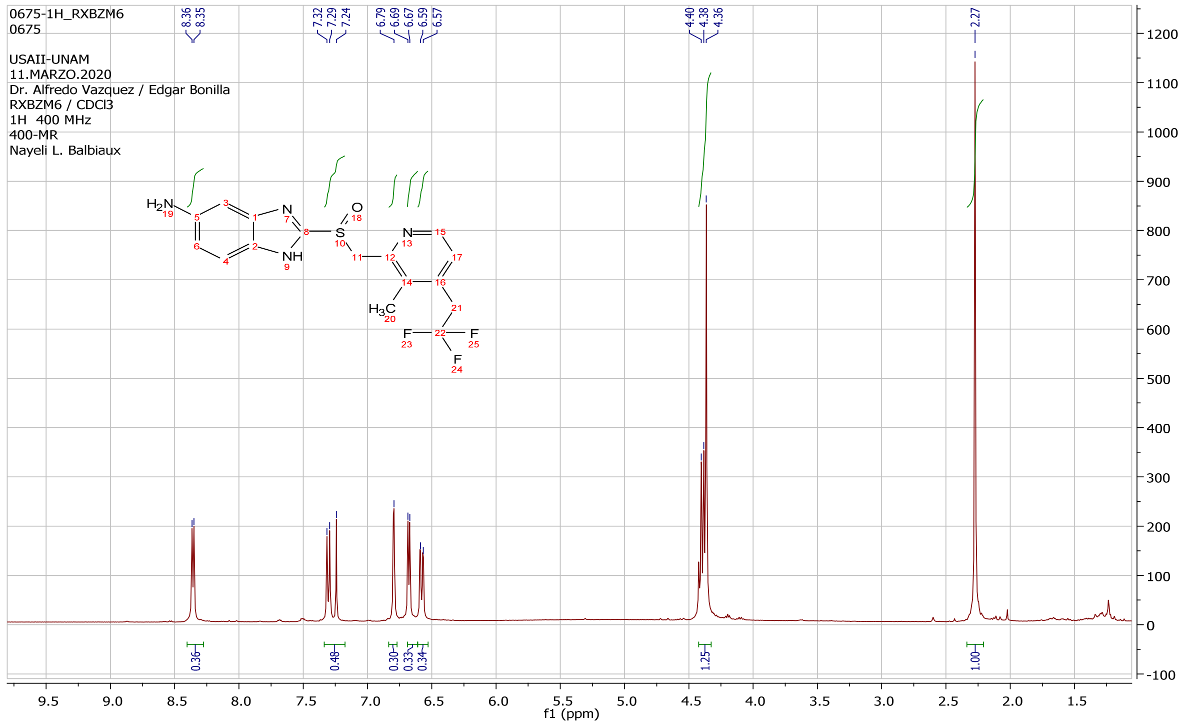

Figure S7. ^{1}H NMR spectrum of compound **H2N-BZM6** in CHCl3-*d6*.

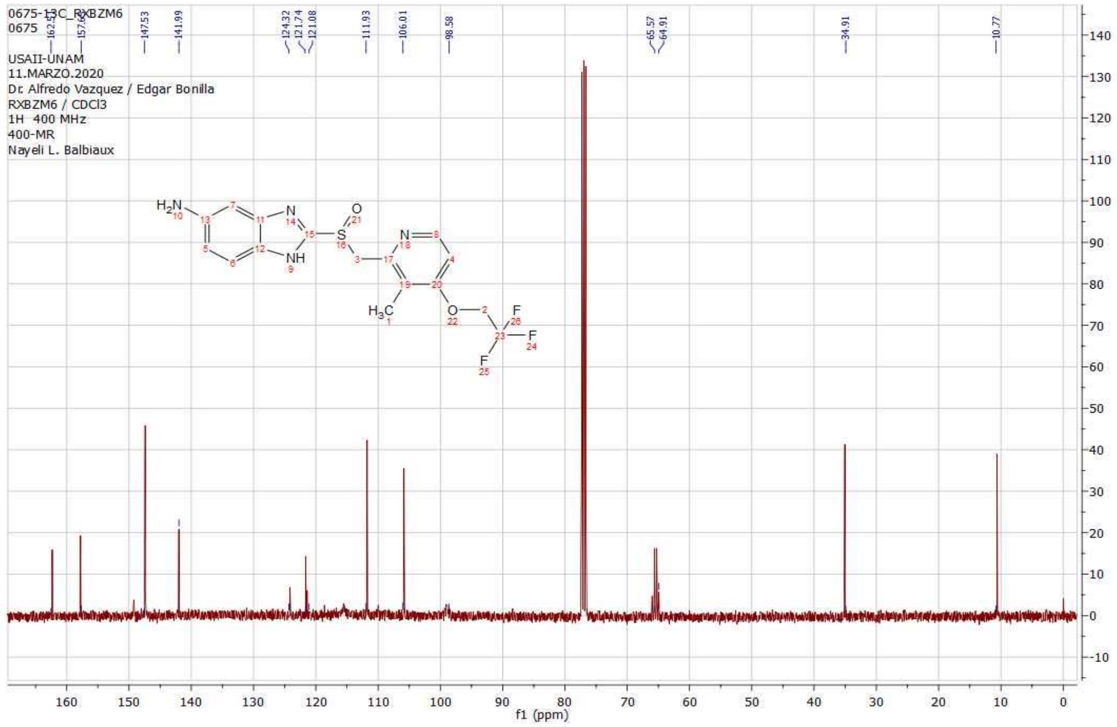

Figure S8. ^{13}C NMR spectrum of compound **H2N-BZM6** in CHCl3-*d6*.

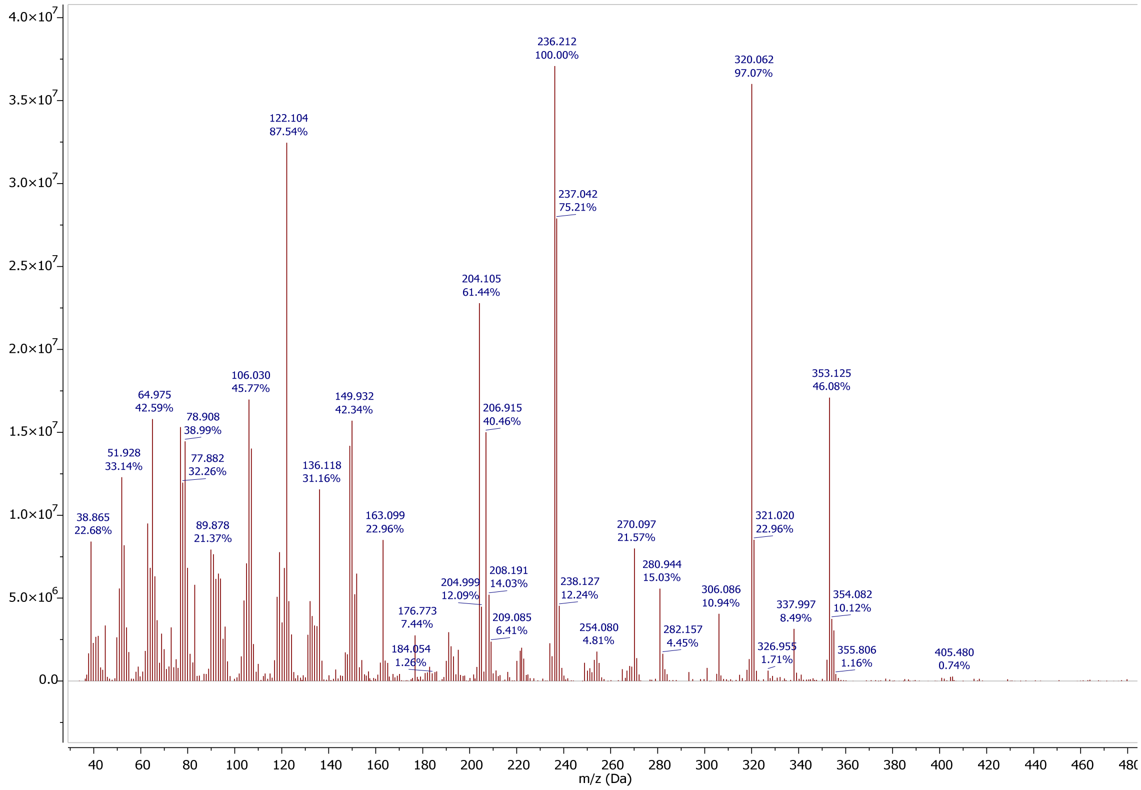

Figure S9. High-resolution mass spectra of compound **H$_2$N-BZM6**

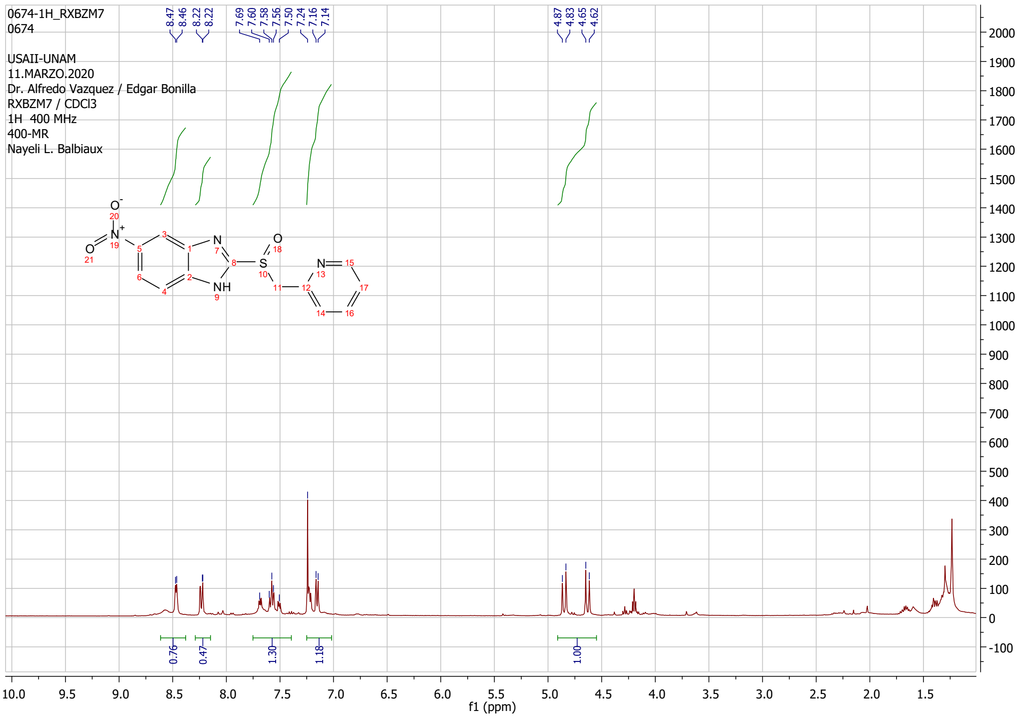

Figure S10. ^{1}H NMR spectrum of compound **O$_2$N-BZM7** in CHCl$_3$-*d6*.

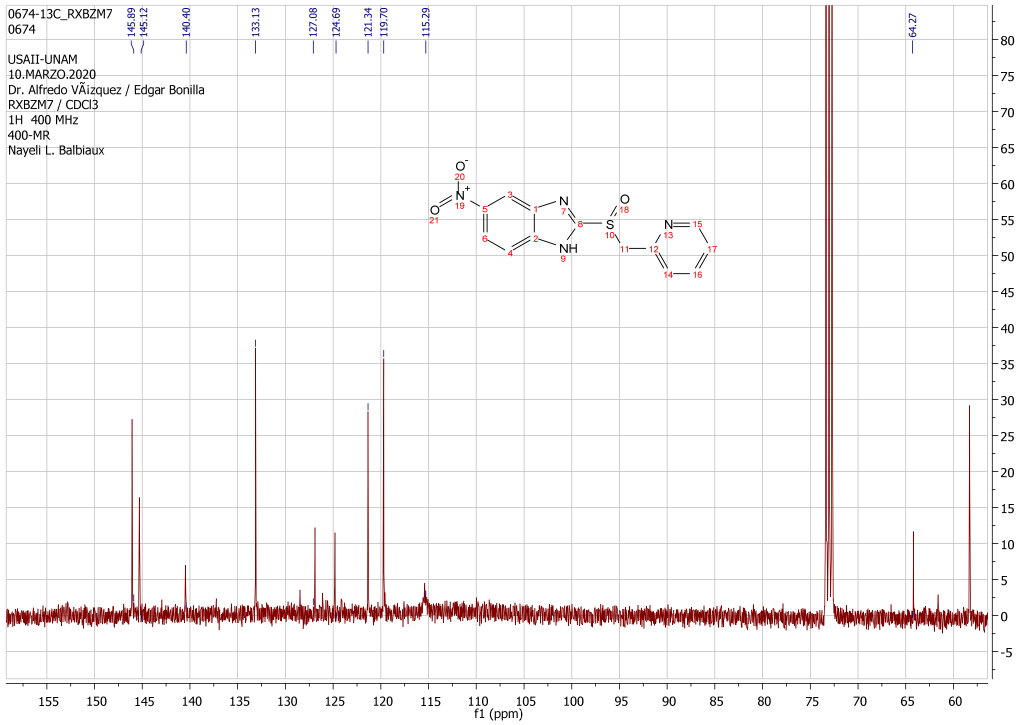

Figure S11. ^{13}C NMR spectrum of compound **O_2N-BZM7** in $CHCl_3$-*d6*.

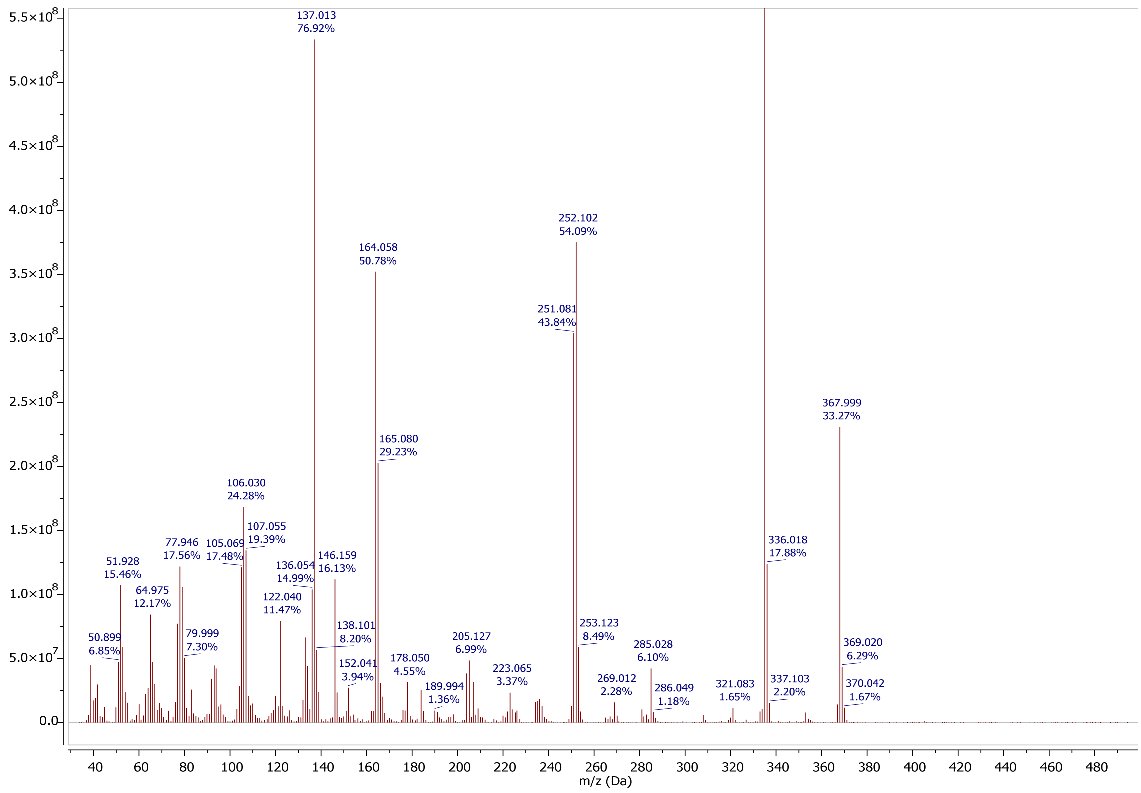

Figure S12. High-resolution mass spectra of compound **O_2N-BZM7**

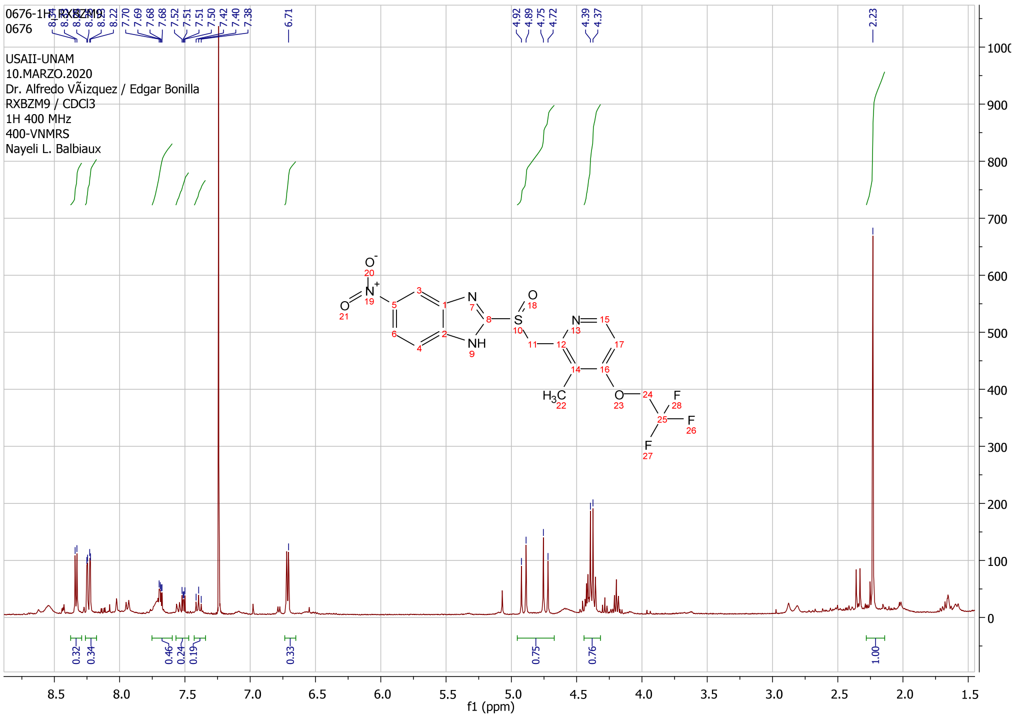

Figure S13. ^{1}H NMR spectrum of compound **O2N-BZM9** in $CHCl_3$-*d6*.

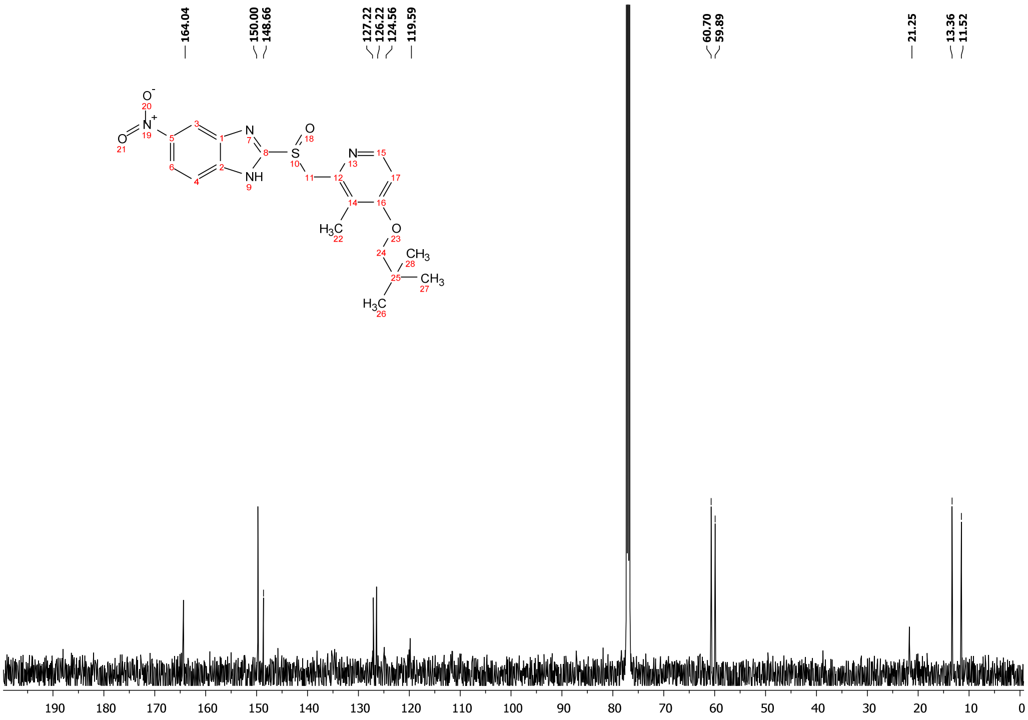

Figure S14. ^{13}C NMR spectrum of compound **O2N-BZM9** in $CHCl_3$-*d6*.

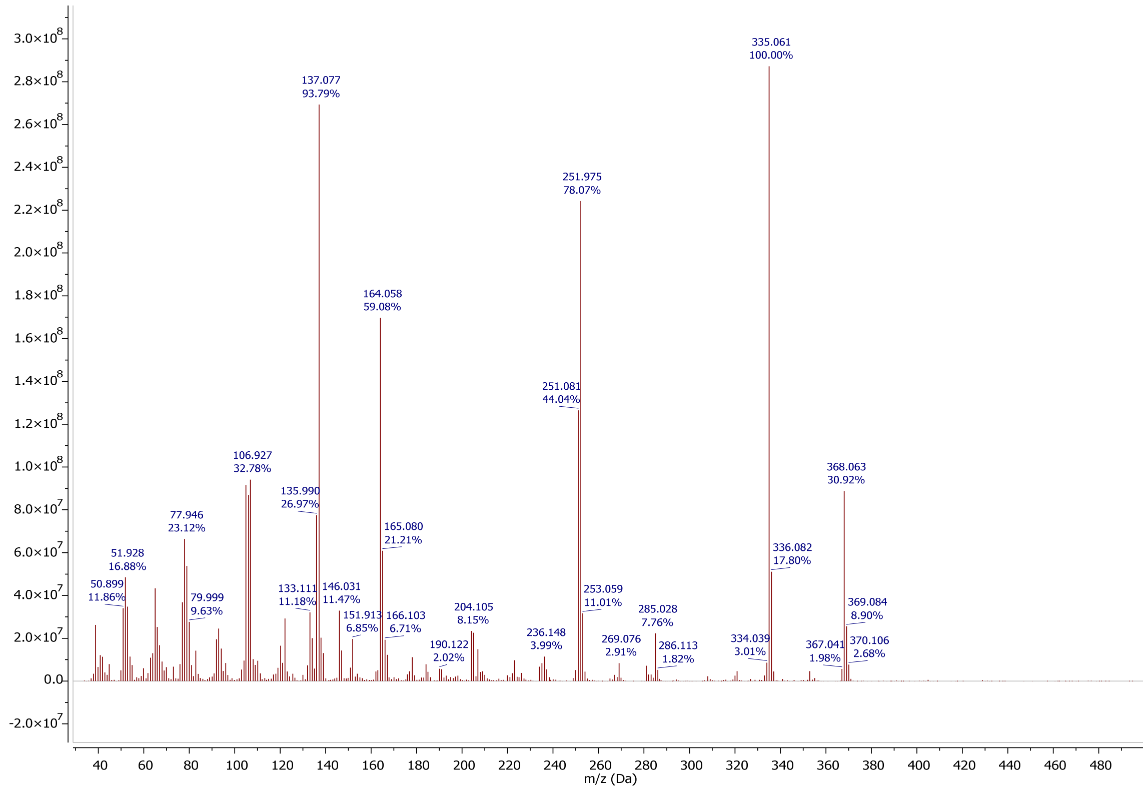

Figure S15. High-resolution mass spectra of compound **O_2N-BZM9**
